# Regulating Glucose Metabolism with Prodrug Nanoparticles for Promoting Photoimmunotherapy of Pancreatic Cancer

**DOI:** 10.1002/advs.202002746

**Published:** 2021-01-04

**Authors:** Fang Sun, Qiurong Zhu, Tianliang Li, Madiha Saeed, Zhiai Xu, Feisheng Zhong, Rundi Song, Manxiu Huai, Mingyue Zheng, Cen Xie, Leiming Xu, Haijun Yu

**Affiliations:** ^1^ Department of Gastroenterology Xinhua Hospital Shanghai Jiaotong University School of Medicine Shanghai 2000092 China; ^2^ State Key Laboratory of Drug Research & Center of Pharmaceutics Shanghai Institute of Materia Medica Chinese Academy of Sciences Shanghai 201203 China; ^3^ School of Chemistry and Molecular Engineering East China Normal University Shanghai 200241 China; ^4^ Yantai Key Laboratory of Nanomedicine & Advanced Preparations Yantai Institute of Materia Medica Shandong 264000 China

**Keywords:** glucose metabolism, immunogenic cell death, pancreatic cancer, photoimmunotherapy, prodrug nanoparticles

## Abstract

The low immunogenicity, insufficient infiltration of T lymphocytes, and dismal response to immune checkpoint blockade therapy pose major difficulties in immunotherapy of pancreatic cancer. Photoimmunotherapy by photodynamic therapy (PDT) can induce an antitumor immune response by triggering immunogenic cell death in the tumor cells. Notwithstanding, PDT‐driven oxygen consumption and microvascular damage can further aggravate hypoxia to exaggerates glycolysis, leading to lactate accumulation and immunosuppressive tumor microenvironment. Herein, a supramolecular prodrug nanoplatform codelivering a photosensitizer and a prodrug of bromodomain‐containing protein 4 inhibitor (BRD4i) JQ1 for combinatory photoimmunotherapy of pancreatic cancer are demonstrated. The nanoparticles are fabricated by host–guest complexation between cyclodextrin‐grafted hyaluronic acid (HA‐CD) and adamantine‐conjugated heterodimers of pyropheophorbide a (PPa) and JQ1, respectively. HA can achieve active tumor targeting by recognizing highly expressed CD44 on the surface of pancreatic tumors. PPa‐mediated PDT can enhance the immunogenicity of the tumor cells and promote intratumoral infiltration of the cytotoxic T lymphocytes. Meanwhile, JQ1 combats PDT‐mediated immune evasion through inhibiting expression of c‐Myc and PD‐L1, which are key regulators of tumor glycolysis and immune evasion. Collectively, this study presents a novel strategy to enhance photoimmunotherapy of the pancreatic cancer by provoking T cells activation and overcoming adaptive immune resistance.

## Graphical abstract

Supramolecular prodrug nanosystem composed of *β*‐cyclodextrin‐grafted hyaluronic acid and reduction‐activatable adamantine‐JQ1/adamantine‐Pheophorbide A conjugates is reported. The prodrug nanoparticle induces immunogenic cell death in the pancreatic tumor cells under near‐infrared laser irradiation, and consequently promoted intratumoral infiltration of the cytotoxic T lymphocytes. Meanwhile, JQ1 relieves glycolysis‐mediated immune evasion and photodynamic therapy‐triggered PD‐L1 overexpression for durable regression of the pancreatic tumor.

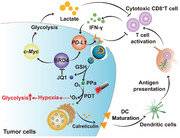

## 1 Introduction

Pancreatic cancer is one of the most aggressive malignancies with a 5‐year survival rate of less than 6%, which is mainly correlated with the late diagnosis and profound radio‐ and chemotherapy resistance.^[^ ^1^, ^2^, ^3^ ^]^ Immune checkpoint blockade (ICB) therapy, as an emerging therapeutic option, has demonstrated promising responses in several subsets of solid tumors, such as melanoma, non‐small cell lung cancer and head and neck squamous cell carcinoma.^[^ ^4^, ^5^, ^6^ ^]^ However, ICB therapy in pancreatic cancer, such as anti‐programmed cell death protein 1 or anti‐cytotoxic T lymphocyte‐associated antigen 4 inhibitors alone or in combination displayed limited clinical benefits.^[^ ^7^ ^]^ Pancreatic cancer is comprised of the highly immunosuppressive tumor microenvironment (ITM), that is, scarce cytotoxic T lymphocytes (CTL) and abundant regulatory T lymphocytes (Tregs), tumor‐associated macrophages , and myeloid‐derived suppressive cells.^[^ ^8^ ^]^ ITM confers immune escape and immune exhaustion in pancreatic cancer, leading to dismal prognosis in clinical studies.^[^ ^9^ ^]^ The infrequent infiltration of CD8^+^ cytotoxic T cells because of low immunogenicity and unique desmoplasia in pancreatic cancer, therefore, remains one of the critical limitations of ICBs.

Photodynamic therapy (PDT) has attracted immense interest because the generated reactive oxygen species (ROS) can drive immunogenic cell death (ICD) in the tumor cells to boost antineoplastic immune responses in vaccine‐based cancer immunotherapy.^[^ ^10^, ^11^, ^12^ ^]^ ROS‐driven ICD stimulates the constellation of alterations in the dying tumor cells, such as calreticulin (CRT) exposure as a “eat‐me” signal, high mobility group box 1 (HMGB1) efflux as “danger” signal and adenosine triphosphate secretion as “find me” signal, to attract and activate antigen‐presenting cells, leading to activation of adaptive immunity.^[^ ^13^, ^14^ ^]^ On the other hand, it has been demonstrated that the activated adaptive immune resistance in response to PDT is positively correlated with tumor recurrence.^[^ ^15^, ^16^ ^]^

PDT utilizes oxygen for generating ROS and thus contribute to the aggravation of intrinsic hypoxic condition in ITM.^[^ ^17^ ^]^ Tumor hypoxia is responsible for metabolic reprogramming, which consumes glucose to provide energy dominantly by glycolysis rather than oxidative phosphorylation.^[^ ^18^ ^]^ Highly glycolytic cancer cells convert pyruvate to lactate, which fuels tumor growth and survival in the hostile microenvironment by activating a series of immunosuppressive pathways.^[^ ^19^, ^20^ ^]^ Meantime, the accumulated lactate poses a major challenge for cancer immunotherapy, where it supports tumor progression while impairing cytokine production and tumor immune surveillance.^[^ ^21^, ^22^ ^]^ In this context, disruption of PDT‐promoted glycolysis can be an effective approach in overcoming tumor immune evasion and tumor recurrence. Oncogene *c‐Myc* as an important transcription factor, which could be involved in regulating tumor glycolysis of pancreatic cancer.^[^ ^23^, ^24^ ^]^ Blockade of *c‐Myc* signaling has potential to triumph over lactate‐mediated CTL inactivation. The development of potent *c‐Myc* antagonists, however, remains a formidable challenge.

**Scheme 1:**
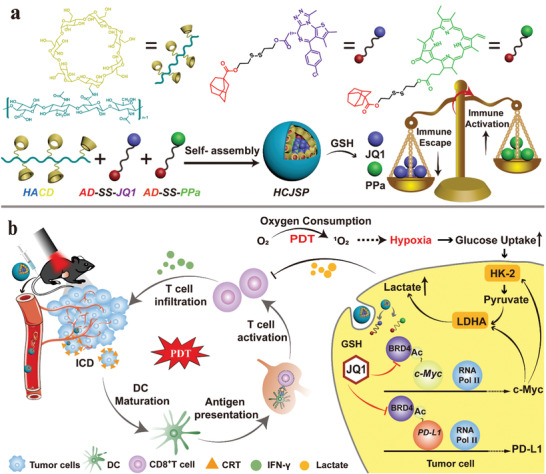
a) Schematic illustration of the HCJSP prodrug nanoparticle prepared via the host–guest interaction between HA‐CD and AD‐SS‐JQ1 and AD‐SS‐PPa. b) Proposed mechanisms of HCJSP‐based combinatory immunotherapy of pancreatic tumor by eliciting immunogenicity and overcoming adaptive immune resistance. HCJSP‐mediated PDT initiates ICD of the tumor cells, promotes DC maturation, and activates the CTLs for tumor regression. Meanwhile, BRD4i JQ1 can relieve PDT‐promoted glycolysis and immunosuppresive tumor microenvironment by impeding the transcription of c‐Myc and the downstream genes of the c‐Myc pathway, including HK‐2 and LDHA. Meanwhile, JQ1 can specifically downregulate IFN‐*γ*‐inducible PD‐L1 expression on the surface of the tumor cells for combating PDT‐inducible adaptive immune evasion.

The critical role of bromodomain and extraterminal protein 4 (BRD4) in epigenetic regulation of *c‐Myc* and programmed death ligand 1 (PD‐L1) transcription has been explored.^[^ ^25^, ^26^, ^27^ ^]^ We thus hypothesized that a combination of BRD4 inhibitor (BRD4i) with PDT might be an ideal approach to boost T cell activation for provoking durable antitumor immunity while overcoming PDT‐induced glycolysis. In an effort to identify the complementary strategy to ensure antitumor immune response activation and glycolysis modulation, we herein presented a supramolecular prodrug‐based nanoplatform for combinatory immunotherapy of pancreatic cancer. The prodrug nanoparticles were fabricated through host–guest interaction between *β*‐cyclodextrin‐grafted hydronic acid (HA‐CD) and a supramolecular prodrug nanosystem to codeliver pyropheophorbide a (PPa) and BRD4i (JQ1) for photoimmunotherapy of pancreatic cancer (**Scheme** **1**a). HA can specifically recognize overexpressed CD44 on the surface of pancreatic tumor cells for facilitating the internalization of supramolecular nanoparticles.^[^ ^28^, ^29^ ^]^ Once internalized into tumor cells, PPa is able to generate ROS under near‐infrared (NIR) laser irradiation, which promotes activation and intratumoral infiltration of CD8^+^ T lymphocytes. JQ1 can be restored from the heterodimer of AD‐SS‐JQ1 in response to the endogenous glutathione (GSH). JQ1, a potent BRDi, can serve as an effective alternative to block the transcription of *c‐Myc* and suppress glycolysis. Meanwhile, JQ1 can combat PDT‐induced immune tolerance by specifically downregulating the expression of PD‐L1 on the surface of tumor cells (Scheme 1b). To the best of our knowledge, this is the first study demonstrating the application of supramolecular nanoparticles for tumor glycolysis and immune microenvironment regulation. The prodrug nanoparticles prepared in a plug‐in style via the host–guest interaction between HA‐CD and the AD‐modified prodrugs can be readily optimized by adjusting the feeding ratio of the prodrug compositions or changing the prodrug types. Therefore, this study might imply a novel strategy for promoting photoimmunotherapy of pancreatic cancer.

## 2 Result and Discussion

### 2.1 JQ1 Alleviates PDT‐Mediated Immune Evasion

**Figure 1:**
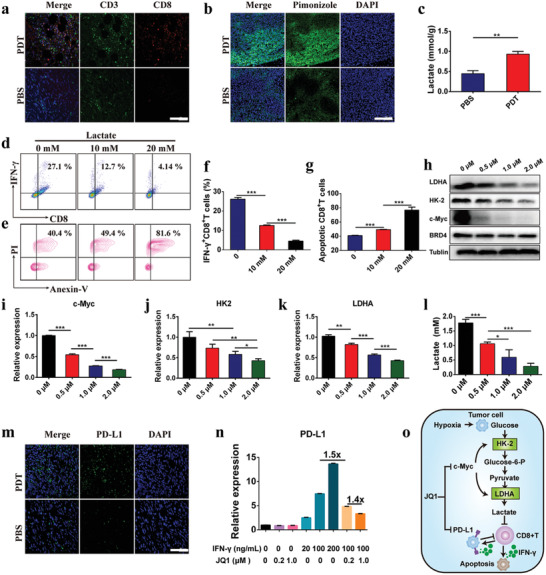
JQ1 ameliorated PDT‐inducible immune evasion of pancreatic cancer. a) Immunofluorescence staining of PDT‐promoted intratumoral infiltration of CD8^+^ T cells in Panc02 tumor‐bearing C57 mice (scale bar = 50 µm). The Panc02 tumor‐bearing C57 mice in PDT group were exposed to 671 nm laser irradiation for 3 min after receiving HCP intravenously at a dose of 5.0 mg kg^−1^ PPa for 24 h. The tumors were harvested and immunostained 7‐days post‐treatment; b) Immunofluorescence assay of PDT‐induced hypoxia in the subcutaneous pancreatic tumors (scale bar = 50 µm); c) Lactate secretion in the pancreatic tumor tissue (n = 3). The tumors were harvested for intratumoral hypoxia and lactate determination 24 h post‐PDT; d–g) Flow cytometric and quantitative analysis of IFN‐*γ* ^+^ CD8^+^ T cells (d and f) and apoptotic CD8^+^ T cells (e and g) after incubated with 10 or 20 mm of lactate for 24 h; h–k) Representative western blot and semi‐quantitative analysis of the expression of c‐Myc (i) and HK‐2 (j), and LDHA (k) after treated with 0.5, 1.0 or 2.0 µm of JQ1 for 24 h; l) Lactate secretion in the cell culture supernatant derived from Panc02 cells incubated with different concentrations of JQ1 for 24 h; m) Immunofluorescence assay of PD‐L1 expression in Panc02‐derived tumor tissue (scale bar = 50 µm); n) Flow cytometric examination of PD‐L1 expression in Panc02 cells treated with IFN‐*γ*/JQ1 alone or combination for 24 h; o) Schematic illustration of mechanism of combination immunotherapy. (mean ± s.d, *p < 0.05, **p < 0.01, ***p < 0.001).

For proof‐of‐concept, we first investigated whether JQ1 can simultaneously abolish PDT‐triggered immune evasion, that is, increase glycolysis and lactate as well as upregulate PD‐L1 expression on the surface tumor cell membrane. To investigate the effect of PDT in vivo, we first prepared a PPa‐loaded supramolecular nanoparticle, which was fabricated by host–guest interaction between HA‐CD and AD‐SS‐PPa (termed as HCP). HA‐CD was prepared by grafting NH_2_‐*β*‐CD onto the backbone of HA via an amide bond (Figures S1 and S2, Supporting Information). AD‐SS‐PPa was synthesized by adamantine‐conjugated heterodimers of PPa linked by a disulfide bond, which was verified by ^1^H NMR spectra and mass spectrum (MS) (Figures S3–S5, Supporting Information). The HCP nanoparticles displayed spherical morphology and averaged hydrodynamic diameter of 97.4 ± 0.2 nm as determined by transmission electron microscopy (TEM) and dynamic light scattering (DLS) (Figure S6, Supporting Information). We confirmed that PDT treatment with HCP can activate CD8^+^ T cells in Panc02‐derived tumor tissue, suggesting that PDT was capable of eliciting an antitumor adaptive immune response (**Figure** **1**a). However, tumor recurrence in the Panc02‐bearing mice was observed post‐PDT.

To optimize the therapeutic outputs of PDT, the underlying mechanism of tumor recurrence post PDT was investigated. It is thought that PDT can confer hypoxia in the tumor by utilizing the oxygen and damaging microvessels. To assess the oxygenation status, the tumor tissues were stained with surrogate hypoxic markers‐pimonidazole‐binding adducts and analyzed by immunofluorescence staining. It was observed that PDT‐mediated oxygen consumption contributes to increasing hypoxia in the tumor tissues (Figure 1b). The elevated hypoxic condition exaggerates glycolysis and ultimate lactate accumulation, that may result in CD8^+^ T cells exhaustion in the tumor microenvironment (Figure 1c).

To explore whether glycolysis metabolite‐lactate accumulation could result in the impairment of CTL function, we detected the apoptosis and cytokine secretion of CD8^+^ T cells after treating with different concentration of lactate in vitro. The CD8^+^ T cells were isolated from C57BL/6 mouse spleen and activated with 50 mm of *β*‐mercaptoethanol, 1.0 µg mL^−1^ of anti‐CD3‐mAb and 1.0 µg mL^−1^ of anti‐CD28‐mAb. The IFN‐*γ* secretion in activated CD8^+^ T cells was impaired after being treated with 10 mm of lactate (Figure 1d,f), while 20 mm lactate remarkably induced apoptosis of the CD8^+^ T cells (Figure 1e,g). This phenomenon is consistent with previous report that lactate could blunt CD8^+^ T activity by hindering the expression of nuclear factor of activated T cells in the T cells, leading to tumor immune evasion.^[^ ^21^ ^]^ To avoid elevated glycolysis‐mediated immune resistance, BRD4i was employed to significantly attenuate the c‐Myc expression as verified in Panc02 cells by western blot. Noticeably, BRD4 expression in the tumor cells remained unimpaired upon JQ1 incubation. In contrast, JQ1 dramatically downregulated BRD4 downstream genes including c‐Myc (Figure 1h,i).

To confirm BRD4i‐mediated *c‐Myc* inhibition, and consequently suppressed lactate generation in Panc02 cells, two key glycolytic enzymes including hexokinase‐II (HK‐2) and lactate dehydrogenase A (LDHA), along with lactate secretion were detected by western blot assay and turned out to be downregulated by JQ1 (Figure 1j–l). PDT‐created immunosuppressive microenvironment was therefore improved through JQ1 by depleting glycolysis and lactate accumulation. Besides, PD‐L1 expression in Panc02 cells was significantly upregulated after stimulating with a series of IFN‐*γ* concentrations that could also contribute to tumor evasion following PDT‐triggered CTL infiltration (Figure 1m,n). Noticeably, Figure 1n displayed that JQ1 suppressed IFN‐*γ*‐ inducible PD‐L1 expression in the tumor cells due to the direct inhibition of BRD4‐mediated PD‐L1 transcription.^[^ ^30^, ^31^, ^32^, ^33^, ^34^ ^]^ The synergistic effect of JQ1 in immunotherapy following PDT‐triggered immune activation was illustrated in Figure 1o.

### 2.2 Preparation and Characterization of the Supramolecular Prodrug Nanoparticles

Given the advantage of JQ1 for glycolysis regulation and CTL activation with PPa, we next sought to construct PPa and JQ1 coloaded supramolecular nanoparticles. We synthesized adamantine‐conjugated heterodimers of JQ1 via a reduction‐liable disulfide spacer, termed as AD‐SS‐JQ1. Structure identification of AD‐SS‐JQ1 was confirmed by ^1^H NMR spectra, MS, and ^13^C NMR spectrum measurements, respectively (Figures S7–S10, Supporting Information). Owing to the host–guest recognition between CD and AD, the supramolecular prodrug nanoparticles, termed as HCJSP, were successfully fabricated by complexing HA‐CD with the reduction‐activatable heterodimer of AD‐SS‐PPa and AD‐SS‐JQ1 at a predetermined JQ1 to PPa molar ratio of 3:1.

**Figure 2:**
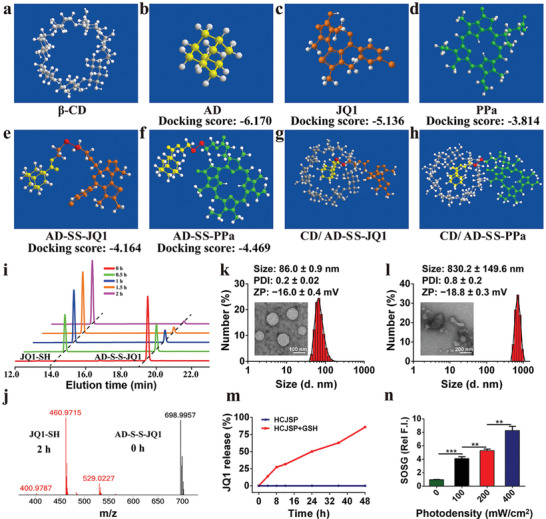
Physicochemical properties of the supramolecular prodrug nanoparticles. a–h) Computer simulation of molecular structure and docking. Binding affinity was analyzed by calculating the docking scores between a) *β*‐CD and b) AD, c)JQ1, d) PPa, e) AD‐SS‐JQ1 or f) AD‐SS‐PPa, respectively; Molecular docking simulation between g) *β*‐CD as a model host and the adamantane moiety of two guests‐ AD‐SS‐JQ1, and h) AD‐SS‐PPa; i) JQ1 release profile of AD‐SS‐JQ1 incubated with 10 mm GSH determined by HPLC, and i) and verified by mass spectrometry; k) TEM images and DLS‐identified size distribution of HCJSP, and l) HCJSP + 10 mm GSH; m) JQ1 release profile of HCJSP nanoparticles in a GSH‐responsive manner; n) Photoactivity examination of HCJSP nanoparticles upon 671 nm laser irradiation at photodensity of 100, 200, or 400 mW cm^−2^ for 30 s.

To discern the host‐guest interaction site between HA‐CD and AD‐SS‐PPa as well as AD‐SS‐JQ1, the tendency of AD/*β*‐CD, JQ1/*β*‐CD or PPa/*β*‐CD complexation was first evaluated by molecular docking simulation and the binding affinity between *β*‐CD and the guest molecules (e.g., AD, JQ1, and PPa) was predicted. The AD/*β*‐CD host‐guest complex displayed a lower docking score of −6.170, in contrast to that of the JQ1/*β*‐CD (−5.136) and PPa/*β*‐CD (−3.814) complexes, indicating *β*‐CD prefers to form stable interaction with AD than JQ1 or PPa (**Figure** **2**a–d). To precisely codeliver JQ1 and PPa, the host–guest interactions between AD‐conjugated heterodimers and HA‐CD were further determined by molecular docking method (Figure 2e–h).

GSH‐mediated cleavage of the disulfide bond in AD‐SS‐JQ1 in the presence of 10 mm GSH was observed by high performance liquid chromatography (HPLC) (Figure 2i). The complete JQ1 release from AD‐SS‐JQ1 post‐2 h incubation with 10 mm GSH was further verified by mass spectrometry (Figure 2j).

To characterize the physicochemical properties of HCJSP, particle size and morphology change upon GSH incubation were monitored with TEM and DLS measurement, respectively. The average hydrodynamic diameter of the spherical nanoparticles increased from 89.0 ± 0.9 to 830.2 ± 149.6 nm upon 24 h incubation with GSH. Along with GSH‐triggered dissociation of the prodrug nanoparticles, their surface charge slightly transformed from −16.0 ± 0.4 mV to −18.8 ± 0.3 mV (Figure 2k,l).

We then investigated the fluorescence spectra of HCJSP, which exhibited a completely quenched fluorescence property as a result of *π*–*π* stacking and Förster resonance energy transfer effect between PPa molecules (Figure S11, Supporting Information). It was worth noting that HCJSP displayed good colloidal stability, especially compared to CJSP, verifying the HA contribution toward the formation and stabilization of supramolecular nanoassemblies (Figure S12, Supporting Information). The superior colloidal stability of HCJSP might facilitate its prolonged blood circulation.

We next examined the reduction sensitivity of the prodrug nanoparticles by assessing GSH‐triggered JQ1 release profile in vitro. Upon 48 h incubation with 10 mm GSH, about 85.8 ± 0.2% of JQ1 was released from HCJSP while kept intact in the absence of GSH (Figure 2m). It was suggested that the cleavage of HCJSP relies on GSH, which guaranteed JQ1 and PPa release under tumor‐specific GSH conditions and thus minimized the systemic side effects.

The ROS generation and photoactivity of HCJSP in solution under 671 nm laser irradiation were detected by Singlet Oxygen Sensor Green. The laser radiation‐triggered ROS generation was observed in a photodensity‐, concentration‐, and time‐dependent manner (Figure 2n and Figure S13, Supporting Information). Thus, we successfully constructed a stimuli‐responsive JQ1 and PPa coloaded nanoplatform with good physicochemical properties to investigate inside into the mechanism underlying the immunosuppressive tumor microenvironment.

### 2.3 Photoactivity and ICD Induction of the Prodrug Nanoparticle In Vitro

**Figure 3:**
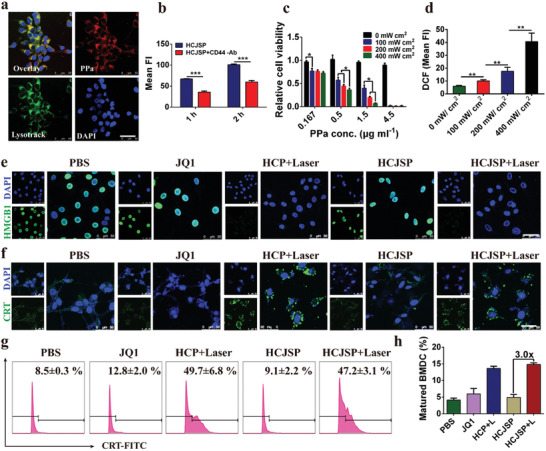
ICD effect of supramolecular nanoparticles‐mediated PDT in the Panc02 cells in vitro. a) CLSM images of cellular uptake of HCJSP post‐24 h incubation (scale bar = 50 µm); b) Flow cytometry detection of cellular uptake of HCJSP pretreated with or without anti‐CD44 antibody; c) Photoactivity of HCJSP in vitro under 671 nm laser irradiation evaluated by CCK8 assay after 24 h treatment; d) Flow cytometry examination determined intracellular ROS generation; e,f) CLSM examination of PDT‐induced surface CRT expression and nuclear HMGB1 efflux in the Panc02 cells in vitro (scale bar = 50 µm); g) The flow cytometric analysis of PDT‐induced CRT exposure of Panc02 cells. Tumor cells were pretreated with HCP or HCJSP at a PPa concentration of 5.0 µm and received 671 nm laser irradiation at 60 mW cm^−2^ for 30 s; h) Flow cytometry detection of PDT‐triggered BMDC maturation in vitro (mean ± s.d, *p < 0.05, **p < 0.01, ***p < 0.001).

Next, we evaluated the intracellular behavior and immune‐activating effect of HCJSP in Panc02 tumor cells in vitro. Confocal laser scanning microscopic (CLSM) analysis demonstrated the endocytosis of HCJSP in Panc02 cells as indicated by colocalization of the prodrug vesicles with lysosomes (**Figure** **3**a). The intracellular fluorescence intensity of PPa showed a time‐dependent increase upon the HCJSP treatment (Figure S14, Supporting Information).

CD44, as HA receptor, is highly expressed in pancreatic cancer cells than adjacent normal tissues and promotes tumor growth and metastasis.^[^ ^28^, ^29^ ^]^ Notably, the cellular uptake of HCJSP slowed down when the Panc02 cells were pretreated with anti‐CD44 antibody (Figure 3b). Hence, HA‐based prodrug nanoparticles are expected to facilitate active tumor targeting to achieve tumor‐specific drug delivery.

Given the superior cellular uptake, Cell Counting Kit‐8 (CCK8) assay was performed to evaluate the cytotoxicity and phototoxicity of HCJSP. The minimum cytotoxicity of the nanoparticles was observed even when the PPa concentration increased up to 4.5 µg mL^−1^. Under 671 nm laser irradiation, the remarkable decrease in cellular viability was dependent on the concentration of HCJSP and photodensity (Figure 3c). Intracellular ROS levels were detected using DCFH‐DA probe and increased in a photodensity‐dependent manner, suggesting HCJSP‐triggered ROS promoted the apoptosis of tumor cells (Figure 3d and Figure S15, Supporting Information).

PDT‐triggered ROS potentially induces ICD under oxidative stress in the tumor cells, which can consequently activate the adaptive antitumor immune response. Membrane exposure of CRT and extracellular efflux of HMGB1 as well‐known hallmarks of ICD were determined in Panc02 cells in vitro. CLSM images demonstrated negligible nuclear HMGB1 in PDT groups that were well‐preserved in PBS and JQ1 groups (Figure 3e), and elevated CRT exposure on the surface of Panc02 cell membrane upon 671 nm laser irradiation (Figure 3f). About 5.8‐ and 5.6‐fold increased CRT exposure in HCP+Laser and HCJSP+Laser treated groups compared to that of the PBS group, respectively, was quantified by flow cytometry (Figure 3g).

The enhanced immunogenicity of pancreatic cancer cells can be employed to potentiate the DC maturation. Bone marrow (BM) cells were extracted from C57BL/6J mice and induced differentiating into BMDCs, which were then coincubated with Panc02 cells (pretreated with 671 nm laser at 60 mW cm^−2^ for 30s). The frequency of matured DCs featured by positive CD11c, CD80, and CD86 was then determined using flow cytometry after 24 h. PDT‐treated HCP and HCJSP groups triggered 3.3‐ and 3.6‐fold higher BMDC maturation than that of the PBS group, respectively, suggesting their potential to elicit antitumor immunity by presenting tumor‐specific antigens to the CD8^+^ T cells (Figure 3h and Figure S16, Supporting Information).

### 2.4 Biodistribution and Antitumor Performance of HCJSP In Vivo

To investigate the intratumoral distribution of the prodrug nanosystem, CJSP and HCJSP nanoparticles were intravenously injected into Panc02 tumor‐bearing nude mice at an identical dose of PPa (5.0 mg kg^−1^). Owing to CD44 receptor‐mediated active targeting capability, HCJSP exhibited superior performance than CJSP at different time points as detected by NIR fluorescence imaging both in vivo and ex vivo.

**Figure 4:**
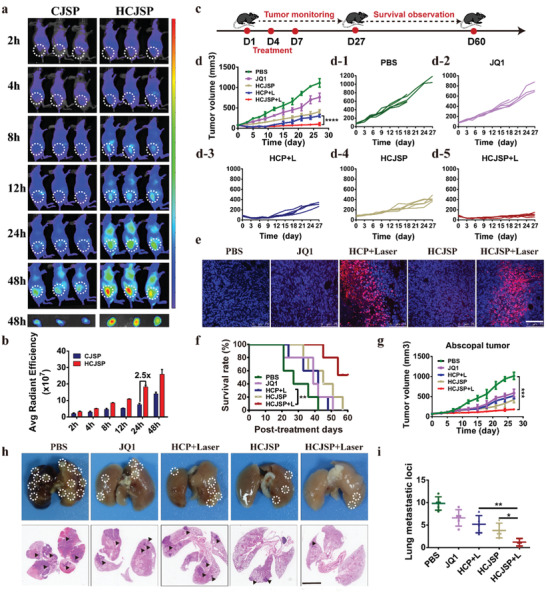
Biodistribution and antitumor effect of the prodrug nanoparticles in vivo. a) IVIS images of CJSP and HCJSP distribution in Panc02 tumor‐bearing mice in vivo; b) Fluorescence imaging and semi‐quantitative analysis of CJSP and HCJSP distribution in the tumor site ex vivo examined at the indicated time points; c) Experimental schedule for HCJSP‐mediated photoimmunotherapy of pancreatic cancer; d) The average and individual tumor growth curves in each experimental group (*n* = 5); The Panc02 tumor‐bearing C57 mice were treated with PBS, JQ1, HCP + Laser, HCJSP, or HCJSP + Laser at an identical JQ1 dose of 15 mg kg^−1^ or PPa dose of 5.0 mg kg^−1^; e) TUNEL staining of the tumor sections at the end of antitumor studies (blue: DAPI; red: TUNEL, scale bar = 100 µm); f) Survival curve of the Panc02‐tumor‐bearing mice following the indicated treatments; g) The average tumor growth curves in each group throughout the observation period (*n* = 5); h,i) Macroscopic and microscopic observation and quantitative analysis of the lung metastatic nodules of in the Pano02 tumor‐bearing mice at the end of the antitumor study. The results were expressed as mean ± s.d, *p < 0.05, **p < 0.01, ***p < 0.001).

The pronounced HCJSP accumulation in the tumor sites was displayed at 4 h post‐injection while 2.5‐fold higher HCJSP accumulation than CJSP at 24 h was attributed to HA‐mediated targeting (**Figure** **4**a,b). Fluorescence imaging ex vivo 48 h post‐injection confirmed the preferred distribution of HCJSP in the tumor sites (Figure S17, Supporting Information). Immunofluorescence staining of the tumor section with anti‐CD31 antibody post 24 h injection of HCJSP further verified its excellent intratumoral penetration in the tumor (Figure S18, Supporting Information). Moreover, the presence of a well‐retained fluorescence signal in the tumor up to 48 h, suggesting HA‐CD44‐mediated active targeting and prolonged blood circulation of HCJSP (Figure 4b).

To assess the antitumor effect of supramolecular prodrug nanoparticles, we prepared a subcutaneous Panc02‐pancreatic tumor model. When the tumor volume reached 100 mm^3^, mice were intravenously treated with PBS, JQ1, HCP+Laser, HCJSP, and HCJSP+Laser at a JQ1 dose of 15.0 mg kg^−1^ or a PPa dose of 5.0 mg kg^−1^ for three times at a time interval of 3 days (Figure 4c). Immunofluorescence examination of tumor tissues revealed the upregulated CRT expression on the surface of the tumor cells 24 h‐post‐treatment in HCP+Laser and HCJSP+Laser group (Figure S19, Supporting Information). Remarkably, although PDT alone dramatically inhibited tumor growth at the beginning, recurrent tumor volume in HCP+Laser group gradually approached to that of HCJSP group, which demonstrated moderate tumor regression during the observation period (Figure 4d). This result could be explained by the hypothesis that the occurrence of adaptive immune resistance induced by PDT prevail over the ICD effect once the treatment disrupted, which could be attributed to PDT‐triggered upregulation of PD‐L1 expression in the tumor cells as we demonstrated previously (Figure 1m,n).^[^ ^11^ ^]^ More prominent apoptosis appeared in the tumor sections of HCJSP+Laser group as determined by terminal deoxynucleotidyl transferase dUTP nick‐end labeling (TUNEL) assay (Figure 4e). Consistently, the antitumor performance was remarkably improved along with the prolonged survival rate because of photoimmunotherapy (Figure 4f). Consequently, we may conclude that JQ1 can triumph over the immune evasion when accompanied by PDT‐triggered ICD because of amplifying robust antitumor efficacy. Indistinct body weight changes and invisible pathological damage of the major organs implied favorable biocompatibility of the prodrug nanoparticles (Figure S20, Supporting Information).

We further established an abscopal tumors model to verify that HCJSP+Laser could trigger a systemic immune response by bilaterally subcutaneous injection of Panc02 cells on C57BL/6 mice. The tumor‐bearing mice were randomly divided into five groups (n = 5), including PBS, JQ1, HCP+Laser, HCJSP, and HCJSP+Laser. Mice received an identical dose (15 mg kg^−1^) of JQ1 or (5.0 mg kg^−1^) PPa. The primary tumor was exposed to 671 nm laser irradiation three times as above. Results showed that HCJSP+Laser exhibited a significant inhibitory effect on abscopal tumors growth (Figure 4g).

To investigate whether HCJSP+Laser elicited the antimetastatic effect, lung metastatic tumor foci were evaluated at the experimental endpoint. The average lung metastatic nodule number in HCP+Laser and HCJSP group was 5.2 ± 1.9 and 3.8 ± 1.6, respectively. However, lung metastasis with 1.2 ± 0.8 was significantly inhibited under the synergistic effects of HCJSP+Laser, suggesting the potent antitumor systemic effect of the prodrug nanoparticles (Figure 4h,i).

### 2.5 HCJSP‐Elicated Combinatory Antitumor Immune Response

**Figure 5:**
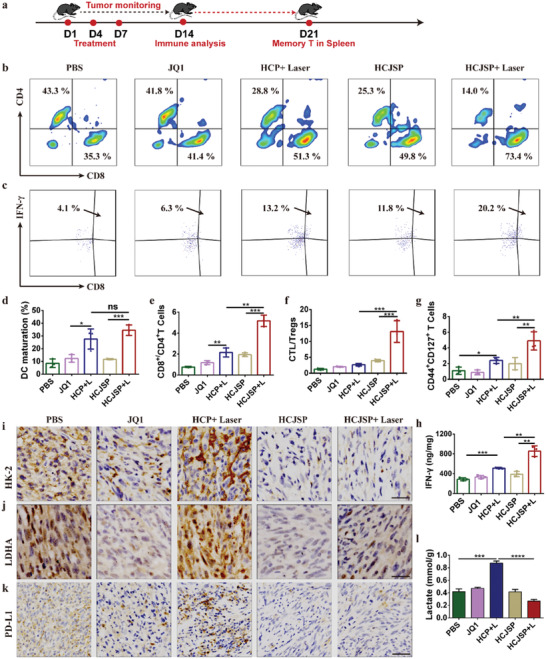
HCJSP‐mediated combinatory immunotherapy of pancreatic cancer. a) Experimental schedule for photoimmunotherapy of HCJSP; b,c) Representative flow cytometric plots of, b) intratumoral infiltrating CD4^+^ and CD8^+^ T cells, and c) intratumoral infiltrating IFN‐*γ* ^+^ CD8^+^ T cells. The tumor‐bearing mice were sacrificed 7‐days post‐treatment for flow cytometric analysis; d) matured DC in the tumor‐draining LNs; e) tumor mass normalized intratumoral infiltration of CD8^+^ T cells to CD4^+^ T cells ratios; f) CD8^+^ T cells to Tregs ratios in TILs; g) the frequency of memory CD8^+^ T lymphocyte in the spleen after 21‐days of treatment; h) intracellular IFN‐*γ* secretion determined by ELISA assay; i–k) IHC examination of i) HK‐2, j) LDHA, and k) PD‐L1 expressions in the tumor tissue at the end of antitumor study (scale bar = 200 µm); l) lactate secretion in the tumor tissue (mean ± s.d, *p < 0.05, **p < 0.01, ***p < 0.001).

Given the decreased recurrence rate of the primary tumors and regression of the abscopal tumors by HCJSP+Laser, to exploit the underlying mechanism of tumor shrinkage, we first harvested the tumor‐draining LNs and measured DC maturation to figure out whether immune activation was involved in the improvement of antitumor performance (**Figure** **5**a). It was observed that HCJSP+Laser group augmented 4.0‐fold higher DC maturation than that of the PBS group, which implicated the potent ICD‐induction of prodrug nanoparticles in vivo. HCJSP+Laser group promoted 1.2‐fold higher DC maturation than HCP+Laser (Figure 5d and Figure S21, Supporting Information).

The maturated DC bridges between ICD of tumor cells and initiation and maintenance of T cell‐mediated response through efficient antigens processing and presentation. Upon DC maturation, intratumoral infiltration of CD8^+^ T lymphocytes was significantly elevated in HCJSP+Laser group. The CD8^+^/CD4^+^ T cells ratio of HCJSP+Laser displayed 2.4‐ and 2.7‐times more than HCP+Laser and HCJSP, respectively (Figure 5b,e). Effector CD8^+^ T cell is a primary source of secreted IFN‐*γ*, which plays an important part in host defense against the tumor. It is worth noting that consistent with the IFN‐*γ* ELISA determination results (Figure 5h), HCJSP+Laser dramatically increased the frequency of IFN‐*γ* ^+^ effector T cells 1.7‐ and 2.0‐fold more efficiently than HCP+Laser and HCJSP, respectively (Figure 5c).

Tregs, one of CD4^+^ T cells subsets, featured by CD25^+^ Foxp3^+^, orchestrate an immune‐privileged status of the immunosuppressive tumor microenvironment.^[^ ^8^ ^]^ The HCJSP+Laser group showed 6.1 ± 2.4% intratumoral infiltration of Tregs, which was 3.9‐times lower than that of the PBS group. Moreover, HCJSP+Laser group presented 4.9‐ and 10.2‐fold higher CD8^+^ T cells to Tregs ratio than those of the HCP+Laser and PBS group, respectively (Figure 5f and Figure S22, Supporting Information), implying that codelivery of PPa and active JQ1 tremendously reversed the ITM in the HCJSP+Laser group.

To elucidate the mechanism underlying the long‐term tumor regression and metastasis inhibition of combinatory therapy, we further examined effective memory T lymphocytes (T_EM_) infiltration in the spleen (CD8^+^CD44^+^CD127^+^) of Panc02 tumor‐bearing C57 mice 21‐day post‐treatment. About 2.0‐ and 2.5‐fold higher frequency of T_EM_ was observed in the spleen after being treated with HCJSP+Laser compared to HCP+Laser and HCJSP, respectively (Figure 5g and Figure S23, Supporting Information). Therefore, the superior lung metastasis inhibition profile of HCJSP+Laser group could be explained by the activation of the long‐term immune memory effect.

The intratumoral secretion of proinflammatory cytokines was examined by ELISA assay. Figure 5h displayed that HCJSP+Laser dramatically induced IFN‐*γ* secretion, which was ≈2.0‐fold more efficient than HCJSP without laser irradiation, verifying the induction of antitumor immune response in the HCJSP+Laser group. Previous studies including ours had identified that intratumoral infiltrating CTLs could trigger PD‐L1 upregulation and induce immunosuppressive tumor microenvironment by secreting IFN‐*γ*.^[^ ^33^, ^34^ ^]^ Immune histochemical (IHC) examination of the tumor sections revealed an obvious decrease in cytoplasmic HK‐2, LDHA, and PD‐L1 expressions post HCJSP+Laser treatment compared to HCP+Laser, suggesting the induction of synergistic immune response by PDT and JQ1‐mediated c‐Myc and PD‐L1 inhibition (Figure 5i–k and Figure S24, Supporting Information), while HCJSP generated 31% less lactate than that of the HCP+Laser group (Figure 5l). We had demonstrated that lactate severely suppressed antitumor immunity by inducing apoptosis of the CTLs (Figure 1g). Therefore, JQ1‐mediated glycolysis inhibition and PD‐L1 downregulation can cumulatively contribute the induction of protective immune response in the HCJSP+Laser group. Taken together, the antitumor and immune data presented in this study consistently verified that our proposed therapeutic strategy is capable of eliciting a potent antitumor immune response in pancreatic cancer while relieving immunosuppression.

## 3 Conclusion

Collectively, we presented a host–guest prodrug nanoparticle‐based combination immunotherapy strategy to overcome the major therapeutic challenges in treating pancreatic cancer. The supramolecular nanoplatform was prepared by integrating HA‐CD with GSH‐activatable AD‐SS‐JQ1 and AD‐SS‐PPa. HA‐based supramolecular nanosystem can achieve prolonged retention and deep tumor penetration owing to its good biocompatibility and active tumor targeting capabilities.^[^ ^35^ ^]^ Besides, the supramolecular prodrug nanoplatform can be evolved by rationally selecting the drug combination and feeding ratio of the therapeutic regimens.^[^ ^36^, ^37^ ^]^ PDT improves the tumor immunogenicity through eliciting ICD cascade yet aggravation of hypoxia hampers the PDT efficacy. JQ1 contributes to overcome PDT‐induced immune resistance by regulating glycolysis and relieving immunosuppression, that is, simultaneous blockade of c‐Myc and PD‐L1 pathway. HCJSP‐mediated photoimmunotherapy significantly inhibited the tumor growth to prolonging survival rate. Moreover, HCJSP+Laser induced systemic immune response and sustained memory effect to prevent tumor recurrence and metastasis. Also, some pilot studies manifest endoscopic ultrasonography (EUS) or endomicroscopy‐guided PDT in pancreatic cancer model appears feasible with minimal side effects.^[^ ^38^, ^39^ ^]^ Furthermore, EUS‐PDT have been successfully conducted in a single‐center, prospective, dose‐escalation phase I clinical study for locally advanced pancreatic cancer.^[^ ^40^ ^]^ Nevertheless, the limitations of subcutaneous syngeneic tumor in differentiating tumor microenvironment and tumor heterogeneity of patients remains a matter of debate. Overall, this study proposed a potential strategy for improving photoimmunotherapy of pancreatic cancer.

## 4 Statistical Analysis

All experiments were carried out for at least three replicates. Data were presented as mean ± standard deviation. Statistical differences were calculated by two‐tailed Student's *t*‐test between two groups and one or two‐way ANOVA among multiply groups performed in Graphpad Prism 6.0. *p* < 0.05 was considered statistically significant.

## Conflict of Interest

The authors declare no conflict of interest.

## Supporting information

Supporting Information
