# Supplementary material for: Regulating Glucose Metabolism with Prodrug Nanoparticles for Promoting Photoimmunotherapy of Pancreatic Cancer
Source: Adv Sci (Weinh). 2021 Jan 4;8(4):2002746. doi: 10.1002/advs.202002746 (PMC7887571; doi:10.1002/advs.202002746)
Supplement: Supplementary file 1 — Supporting Information [file ADVS-8-2002746-s001.pdf]

© 2020 Wiley-VCH GmbH

ADVANCED SCIENCE
Open Access

Supporting Information

for *Adv. Sci.,* DOI: 10.1002/advs.202002746

Regulating Glucose Metabolism with Prodrug Nanoparticles for Photoimmunotherapy of Pancreatic Cancer

Fang Sun, Qiurong Zhu, Tianliang Li, Madiha Saeed, Feisheng Zhong, Rundi Song, Manxiu Huai, Leiming Xu Zhiai Xu, Mingyue Zheng, Cen Xie, Haijun Yu**

Supporting Information

For

Regulating Glucose Metabolism with Prodrug Nanoparticles for Photoimmunotherapy of Pancreatic Cancer

Fang Sun[1,2,#], Qiurong Zhu[2,#], Tianliang Li[2], Madiha Saeed[2], Feisheng Zhong[2], Rundi Song[2], Manxiu Huai[1], Leiming Xu[1,*], Zhiai Xu[3], Mingyue Zheng[2], Cen Xie[2], Haijun Yu[2,4,*]

F. Sun, M. Huai, Prof. L. Xu

Department of Gastroenterology, Xinhua Hospital, Shanghai Jiaotong University School of Medicine, Shanghai, 2000092, China

E-mail: xuleiming@xinhuamed.com.cn

Q. Zhu, Dr. M. Saeed, Dr. T. Li, F. Zhong, R. Song, Prof. M. Zheng, Prof. C. Xie, Prof. H. Yu

State Key Laboratory of Drug Research & Center of Pharmaceutics, Shanghai Institute of Materia Medica, Chinese Academy of Sciences, Shanghai 201203, China

E-mail: hjyu@simm.ac.cn

Prof. Z. Xu

School of Chemistry and Molecular Engineering, East China Normal University, Shanghai 200241, China

Prof. H. Yu

Yantai Key Laboratory of Nanomedicine & Advanced Preparations, Yantai Institute of Materia Medica, Shandong 264000, China.

Table of Contents

**Experimental section**	Page 4-11
**Figure S1.**	Page 12
**Figure S2.**	Page 13
**Figure S3.**	Page 14
**Figure S4.**	Page 15
**Figure S5.**	Page 15
**Figure S6.**	Page 16
**Figure S7.**	Page 17
**Figure S8.**	Page 18
**Figure S9.**	Page 19
**Figure S10.**	Page 20
**Figure S11.**	Page 21
**Figure S12.**	Page 22
**Figure S13.**	Page 23
**Figure S14.**	Page 24
**Figure S15.**	Page 25
**Figure S16.**	Page 26
**Figure S17.**	Page 27
**Figure S18.**	Page 28
**Figure S19.**	Page 29
**Figure S20.**	Page 30
**Figure S21.**	Page 31
**Figure S22.**	Page 32
**Figure S23.**	Page 33
**Figure S24.**	Page 34

Experimental section

Materials. Sodium hyaluronic acid (HA, *Mw* = 36 kDa) was purchased from Bloomage Freda Biopharm Co., Ltd (Shandong, China). Pyropheophorbide a (PPa) and 6-monodeoxy-6monoamino β-cyclodextrin (NH_2-β-CD, 98%) were purchased from Dibai Chem Tech Co., Ltd (Shanghai, China). JQ1 was purchased from Selleck Chem Co., Ltd (Shanghai, China). Triphosgene and bis (2-hydroxyethyl) disulfide were purchased from TCI (Shanghai, China). N-(3-(dimethylamino)-propyl)-N-ethylcarbodiimide hydrochloride (EDCI), 1-hydroxybenzotriazole anhydrous (HOBT), triethylamine (TEA), 4-dimethylaminopyridine (DMAP) and trifluoroacetic acid (TFA) were purchased from J&K Scientific Ltd (Beijing, China). Glutathione (GSH), 2′,7′-dichlorofluorescein diacetate (DCFH-DA) were all purchased from Sigma-Aldrich (Shanghai, China). Singlet Oxygen Sensor Green (SOSG) and Cell Counting Kit-8 were purchased from Dalian Meilun Biotech CO., Ltd (Dalian, China).

The mouse lymphocyte separation medium was obtained from Dakewe Biotech (China). Antibodies against CD11c, CD80, CD86, CD45, CD3, CD4, CD8, IFN-γ, CD25, Foxp3, CD44 and CD127 for flow cytometry were all obtained from BD Biosciences (USA). Antibodies against PD-L1 for western blot were ordered from CST (USA). Antibodies against Tublin, BRD4, c-Myc, HK-2, LDHA, calreticulin and HMGB1 were all ordered from Abcam (UK). IFN-γ, GM-CSF, IL-4, and ELISA kit of TNF-α were all purchased from Neobioscicence Technology Co., Ltd (USA). Anti-CD3 mAb and anti-CD28 mAb were purchased from Biolegend (USA). Magnetic separation, anti-CD8 beads, Tissue Dissociator were obtained from Miltenyi Biotec (Germany).

Cell lines and animals. Panc02 murine pancreatic tumor cells were purchased from the cell bank of the Chinese Academy of Sciences (Shanghai, China). Cells were maintained in DMEM (Gibco) supplemented with 10% fetal bovine serum (FBS), 2 mM of L-glutamine, 1 mM of

sodium pyruvate, 0.1 mM of non-essential amino acids, and 1% Penicillin-Streptomycin at 37 °C in 5% CO_2.

For *in vivo* studies, mice (3-4-week old) were obtained from the Shanghai Experimental Animal Center (Shanghai, China). All mice were kept under the pathogen-free condition and used by following the animal experimental guidelines approved by the Institute of Animal Care and Use Committee, Shanghai Institute of Materia Medica, Chinese Academy of Sciences.

Synthesis of HA-CD, AD-SS-JQ1, and AD-SS-PPa. HA-CD was synthesized by grafting NH_2-β-CD onto the backbone of HA and purified by dialysis against DI water. The final product was obtained by lyophilization. The CD grafting ratio was determined by ^{1}H-NMR spectrum examination.

To synthesize PPa-SS-OH, 400 mg of PPa (F_W 534.7 Da, 1.0 eq), 428.7 mg EDCI (F_W 191 Da, 3.0 eq), and 303.2 mg of HOBT (F_W 135.1 Da, 3.0 eq) was dissolved in 20 mL of anhydrous DMF under Ar and stirred under ice bath for 2h to activate the carboxyl group. The above solution was slowly added with 270 μL anhydrous DMF solution of 2-hydroxyethyl disulfide (F_W 154.3 Da, 2.0 eq), the reaction was continued for 24 h. The product was purified using column chromatography with 20:1 mixed solvent of dichloromethane/methanol as the eluent to obtain PPa-SS-OH as a dark green powder, yield 91% (**Figure S1**).

To synthesize AD-SS-PPa, 454.4 mg of PPa-SS-OH (F_W 670.3 Da, 1.0 eq), 269.4 mg of adamantoyl chloride (F_W 198.7 Da, 2.0 eq) and 82.7 mg of DMAP (F_W 122 Da, 1.0 eq) were dissolved in 20 mL of anhydrous dichloromethane, stirred it in dark at room temperature, and reacted for 24 h. The product was purified by column chromatography with 100:1 mixed solvent of dichloromethane/methanol as the eluent, yield = 85% (**Figure S1**).AD-SS-JQ1 was synthesized by coupling JQ1 (BRD4 inhibitor) with adamantane via a disulfide bond. Briefly, 437 mg of JQ1-COOH, 626 mg of EDCI (F_W 191 Da, 3.0 eq), 400.2 mg of DMAP (F_W 122 Da, 3.0 eq) and 540 μL of DIEA (F_W 129 Da, 3.0 eq) were dissolved in 20 mL of anhydrous DMF

for 2 h to activate the carboxyl group. Afterward, 270 μL of 2-hydroxyethyl disulfide (Fw 154.3 Da, 2.0 eq) dissolved in anhydrous DMF, were slowly dropped to a round-bottom flask and stirred at room temperature for 24h. The raw product was purified by column chromatography with dichloromethane/methanol (10:1) as the eluent to obtain JQ1-SS-OH as a light yellow powder, yield = 95% (**Figure S5**).

To synthesize AD-SS-JQ1, 536 mg of JQ1-SS-OH (Fw 536.1 Da, 1.0 eq), 400 mg of adamantoyl chloride (Fw 198.7 Da, 2.0 eq), and 122 mg, of DMAP (Fw 122 Da, 1.0 mmol, 1 eq) were dissolved in 10 mL of anhydrous dichloromethane under argon protection. The reaction was stirred at room temperature under dark for 24 h. The raw product was purified by column chromatography with 100:1 of dichloromethane/methanol to obtain AD-SS-JQ1 as a light yellow powder, yield = 85% (**Figure S5**).

Fabrication and characterization of the prodrug nanoparticle. The supramolecular prodrug nanoparticles were prepared by the host-guest self-assemble between β-CD and adamantane. Typically, HA-CD (0.4 mg, 0.20 μmol), AD-SS-JQ1 (0.105 mg, 0.15 μmol) and AD-SS-PPa (0.042 mg, 0.05 μmol) were dissolved in 100 μL of DMSO and added into 1 mL aqueous solution of HA-CD during sonication under an ice bath. The purified nanoparticles termed HCJSP were obtained after the removal of free components and DMSO using dialysis with deionized water overnight (MWCO 3.5 kDa). The nanoparticles loaded in ultrafiltration tubes (MWCO 3.0 kDa) were concentrated using ultracentrifugation before the animal study. Similar procedures were followed to prepare CJSP by integrating with β-CD instead of HA-CD and HCP by complexing HA-CD with AD-SS-PPa.

The hydrodynamic diameter, polydispersity (PDI) of size distribution, surface ζ-potential, and morphology of the HCJSP, HCP, and CJSP nanoparticles were examined using dynamic light scattering (DLS) and transmission electron microscopy (TEM). The photoactivity of the nanoparticles was evaluated by using SOSG as the fluorescence ROS probe.

The JQ1 release profile of HCJSP was evaluated by HPLC measurement. Briefly, the prodrug nanoparticles were incubated with 10 mM of GSH at 37 °C. JQ1 release from the nanoparticles was then monitored by using HPLC and mass spectrum at a time interval of 0.5, 1.0, 1.5, and 2.0 h.

Molecular docking. The molecular docking was performed with Glide in Schrödinger 2015 software (USA). The molecular structure of β-CD was supplied by the Protein Preparation Wizard. The hydrogen bond network of β-CD was optimized in the OPLS2005 force field to reorient side-chain hydroxyl and reduce potential steric collision. AD, JQ1, and PPa were obtained from ChemDraw and optimized by LigPrep panel in Maestro of Schrödinger. The docking scores were calculated to predict the host-guest affinity.

Cellular uptake of prodrug nanoparticles *in vitro*. Panc02 cells grown in a 24-well plate were incubated with HCJSP at the desired time points (*i.e.*, 2, 4, 8, 12 and 24 h). The intracellular fluorescence intensity of PPa was then examined by flow cytometric measurement and visualized by CLSM. To verify the active tumor targeting profile of HA, the Panc02 cells were pretreated with an anti-CD44 antibody for 30 min followed by the HCJSP incubation.

Phototoxicity of the prodrug nanoparticles. CCK-8 assay was performed to evaluate the cell viability. Panc02 cells were seeded and maintained for 24 h in 96-well plates at a density of 5000 cells/well. After incubating with different concentrations of the prodrug nanoparticles (*i.e.*, 0.17, 0.5, 1.5 and 4.5 μg/mL) for 12 h, the cells were then illuminated with a 671 nm laser for 30 s at photodensity of 100, 200 or 400 mW/cm^2. The cells were cultured for an additional 24 h and examined by CCK8 assay.

PDT-induced ROS generation *in vitro*. To monitor intracellular ROS production *in vitro*, Panc02 cells with 60%-70% confluence were co-incubated with HCJSP involving 5 μM PPa for 12 h. The cells were washed and supplemented with Dichlorofluorescein (DCFH, 10 μM) as a ROS probe for 20 min under darkness before exposure to 671 nm laser (100, 200 or 400

mW/cm^2) for 30s, respectively. The cells were then harvested and underwent flow cytometric measurement (Ex/Em = 480/550 nm). For CLSM examination, the cells were subjected to 200 mW/cm^2 laser for 30s and then stained with DAPI before observation.

Western blot assay. The cells were collected and lysed with RIPA lysis buffer containing 1% protease inhibitor. Equal amounts of protein were loaded and separated using SDS-PAGE gels, and then transferred to PVDF membranes. Blocking with 5% BSA in TBST for 1h, membranes were conjugated to primary antibodies of BRD4 (1:1000), c-Myc (1:1000), HK-2 (1:1000), LDHA (1:1000), Tubulin (1:1000) overnight at 4 °C. After incubation with HRP-conjugated secondary antibodies for 1h at room temperature, signal visualization was carried out using gel imager (Bio-Rad Laboratories, USA) and analyzed by Image J (NIH, USA).

Determination of immunogenic cell death *in vitro*. The CRT exposure and nuclear HMGB1 efflux were detected to verify the ICD effect of HCP and HCJSP on Panc02 cells. In brief, cells were seeded on 24-well plates at a density of 4 x 10^5 cells/well and cultured for 24 h. Followed by the incubation with HCP or HCJSP at an identical PPa concentration of 5.0 μM for 12 h. The cells were then irradiated with 671 nm (60 mW/cm^2) for 30s. For detecting surface CRT expression, the cells were stained with Alexa488-conjugated monoclonal secondary antibody for an additional 30 min under dark condition after incubation with anti-CRT primary antibody for 30 min. The cells were then analyzed by flow cytometry.

To visualize surface CRT and nuclear HMGB1 by immunofluorescent staining, cells were inoculated into 24-well plates with prepared slides and repeated the same experimental conditions. The adherent cells were washed twice with PBS, then fixed with 4% paraformaldehyde for 20 min. To detect HMGB1 efflux, the cells were permeabilized in 0.1% Triton X-100 for 5 min and then blocked in 5% FBS for 1 h at room temperature. The HMGB1 and CRT staining were processed as described above. After staining with DAPI for 5 min, the cells were examined by CLSM (Leica, Germany).

DC maturation *in vitro*. To validate whether the pre-treated tumor cells can promote DC maturation *in vitro*, we extracted the bone marrow-derived monocytes from 6-8 weeks old C57BL/6 mice. Monocytes were cultured in complete RPMI containing mouse recombinant GM-CSF (20 ng/mL) and IL-4 (20 ng/mL) for 6 days to differentiate into $CD11c^{+}$ BMDCs. BMDCs were stimulated with treated tumor cells for 24 h, then stained with fluorescence-labeled antibodies against CD11c, CD80, and CD86. CD80/CD86 double-positive DCs gated by $CD11c^{+}$ were recognized as maturated DCs.

Tumor targeting and biodistribution of the prodrug nanoparticles *in vivo*. Pacn02 tumor-bearing mice were intravenously injected with PPa-loaded nanoparticles at a dose of 5.0 mg/kg. Biodistribution of nanoparticles *in vivo* at the different time points (2, 4, 8, 12, 24, and 48 h) was determined by IVIS Lumina II In Vivo Imaging System (Caliper LifeSciences)) (Ex/Em = 670 nm/720 nm). The major organs (heart, liver, spleen, lung, and kidney) and tumors were collected and likewise imaged *ex vivo* at 8, 12, 24, and 48 h post-administration. Accumulation of nanoparticles within tumors was assessed by the relative Avg Radiant Efficiency.

Antitumor study and biosafety assay *in vivo*. To explore the antitumor effect because of the synergism between PDT and JQ1, the Panc02 tumor-bearing C57BL/6 mice model was established by subcutaneously injection of 5×10^{6} Panc02 cells on the right flank of mice. When the tumor volume reached 100 mm^{3}, the mice were randomly divided into the following groups (n = 5): PBS, JQ1, HCP+Laser, HCJSP and HCJSP+Laser. The mice were intravenously injected with JQ1, HCP or HCJSP at an equal dose of PPa (5.0 mg/kg) and JQ1 (15 mg/kg). Twenty-four h later, the tumors in laser groups were irradiated with a 671 nm laser at 200 mW/cm^{2} for 3 min. The intervention was repeated for three times every three days. The tumor size and body weight were recorded every other day during the observation period. The tumor volume was determined following the formula: Tumor volume = length × width × width/2 (length, the longest dimension; width, the shortest dimension).

At the endpoint of antitumor study, the major organs (heart, liver, spleen, lung, and kidney) and tumors were preserved for HE or TUNEL staining to examine biosafety and apoptosis of tumor cells. Lung metastasis was evaluated by gross and microscopic appearance. Overall survival was determined in a separate experiment.

For abscopal antitumor experiments, mice were equivalently inoculated with tumor cells on the left flank simultaneously with the right flank tumor. Unilateral tumors were treated as mentioned above. Bilateral tumors growth was measured for a total of 30 days to assess the distant antitumor efficacy.

Immune assay *in vivo*. To investigate the induction of protective antitumor immune effect, tumors and lymph nodes were harvested 7-days post the final treatment. Briefly, tumors were cut into small pieces after weighing, then suspended in RPMI160 with indicated digestive enzymes and dissociated by the gentle MASC^{TM} dissociator (Miltenyi, German). The single-cell suspension was obtained by filtering through 70 μm filters. Tumor-infiltrating lymphocytes (TILs) were further separated and enriched by mouse lymphocyte separation medium. After counting, the TIL suspension stained with anti-CD45-APC, anti-CD3-PerCP-Cy5.5, anti-CD4-FITC, and anti-CD8-PE to quantify the subpopulations of $CD4^+$ and $CD8^+$ T cells. For detection of cytotoxic lymphocyte subsets, i.e., (CTL, IFN-$\gamma^+$$CD8^+$ T cells) and regulatory T cells (Treg, $CD25^+$$Foxp3^+$ $CD4^+$T cells), the single cell suspension were pre-treated with permeabilization, and stained with anti-CD3-PerCP-Cy5.5, anti-CD8-PE and anti-IFN-γ-FITC antibodies, anti-CD3-PerCP-Cy5.5, anti-CD4-FITC, anti-CD25-APC and anti-Foxp3-PE, respectively. All samples were incubated with specific antibodies for 30 min at 4 °C in the dark, then measured by FACS fortessa flow cytometry (Bioscience) and analyzed with FlowJoV10 software. Data were presented after normalization according to the tumor weight and lymphocytes counting.

To determine DC maturation *in vivo*, lymph nodes were ground into a single cell suspension and stained with anti-CD11c-FITC, anti-CD80-PE, and anti-CD86-PE-Cy7. Matured DCs ($CD11c^+CD80^+CD86^+$) were then analyzed by flow cytometry.

To investigate the memory T lymphocytes in spleens 21-days post-treatments, the single-cell suspension of spleens was collected by filtering after gentle grinding and purified by erythrocyte lysis buffer. Memory T cells ($CD44^+CD127^+CD8^+$) stained with anti-CD8-FITC, anti-CD44-PerCP-Cy5.5, and anti-CD127-PE were detected using flow cytometry.

Hypoxia detection in tumor tissue. To investigate the hypoxia status of tumor tissues, Hypoxyprobe™-1 (pimonidazole HCl) solution at a dosage of 60 mg/kg was i.v. injected into the Panc02-bearing mice. The tumors were collected and immunostained with FITC-MAb1 after 1 h of indicated treatment. Hypoxia status in TME was determined based on fluorescence from the reduction of pimonidazole.

Immunosuppressive effects on $CD8^+$ T cells exerted by lactate *in vitro*. Naive $CD8^+$ T cells derived from mice spleen were prepared using a Naive $CD8^+$ T cell Isolation kit. Naive $CD8^+$ T cells (1.0 x10^6/well) were seeded into 24-well plate and stimulated with immobilized anti-CD3 mAb (1 μg/ml) and anti-CD28 mAb (1 μg/ml) for 2 days. After treatment with 10 mM, 20 mM lactate for 24 h, the pro-apoptotic effect on activated $CD8^+$ T cells was evaluated by Annexin V/PI Apoptosis Detection Kit (Yeasen) according to the manufacturer's instructions. IFN-γ^+CD8^+ T cells were then stained and detected using flow cytometry.

Statistical analysis. All experiments were carried out for at least three replicates. Data were presented as mean ± standard deviation (SD). Statistical differences were calculated by two-tailed Student's t-test between two groups and one or two-way ANOVA among multiply groups performed in Graphpad Prism 6.0. $p < 0.05$ was considered statistically significant.

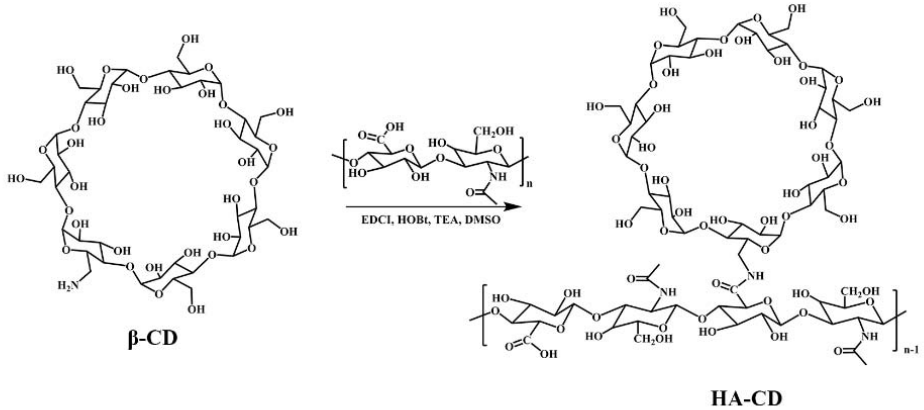

Figure S1. Synthetic route of HACD.

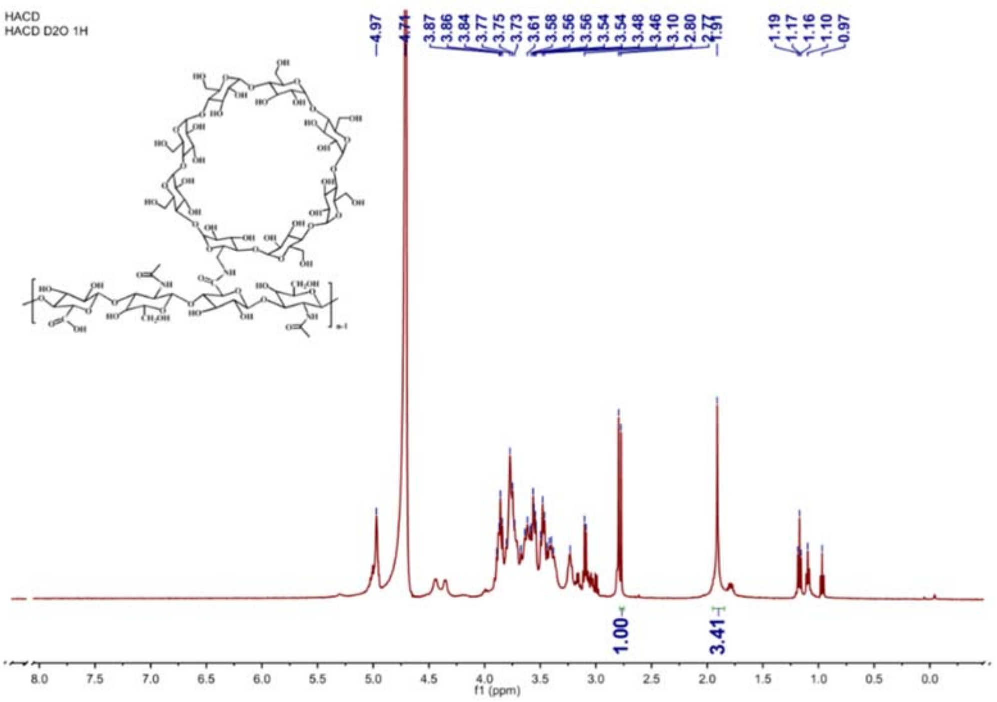

Figure S2. ^{1}H-NMR spectrum of HACD.

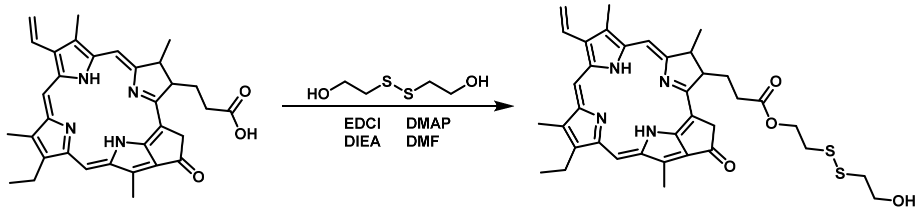

D

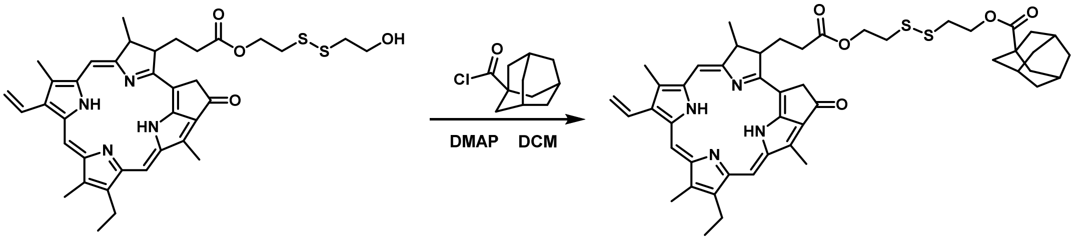

E

Figure S3. Synthetic route of AD-SS-PPa.

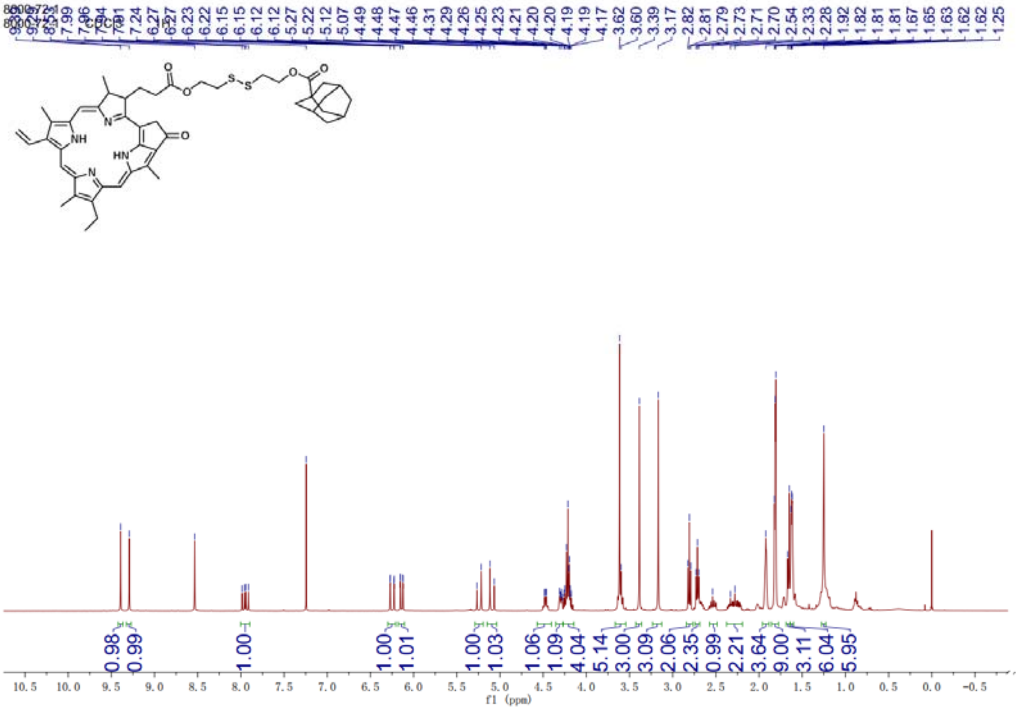

Figure S4. The ^{1}H-NMR spectra of AD-SS-PPa.

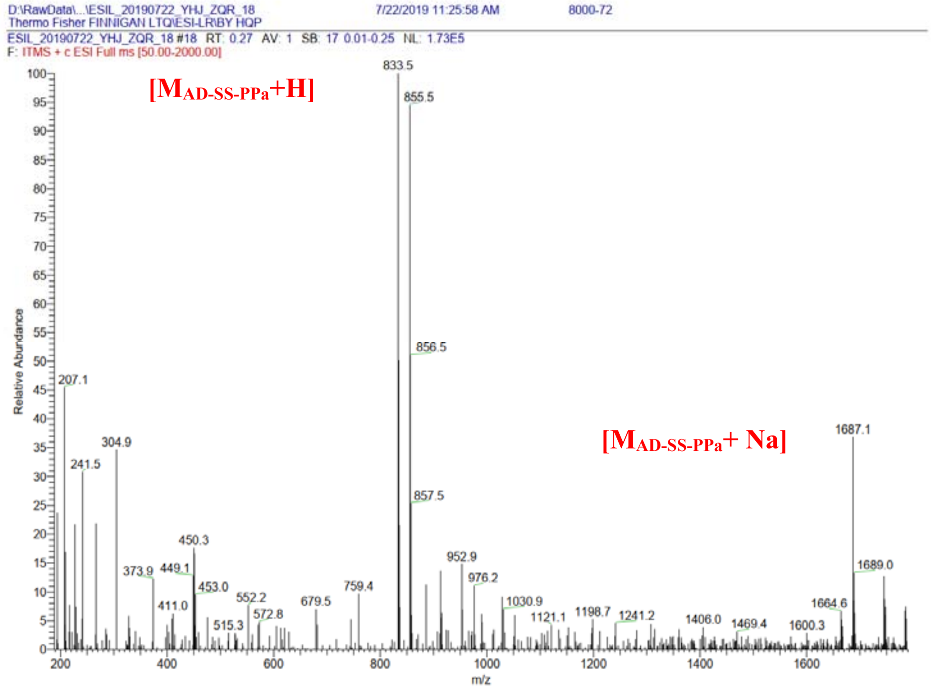

Figure S5. The ESI-MS spectrum of AD-SS-PPa.

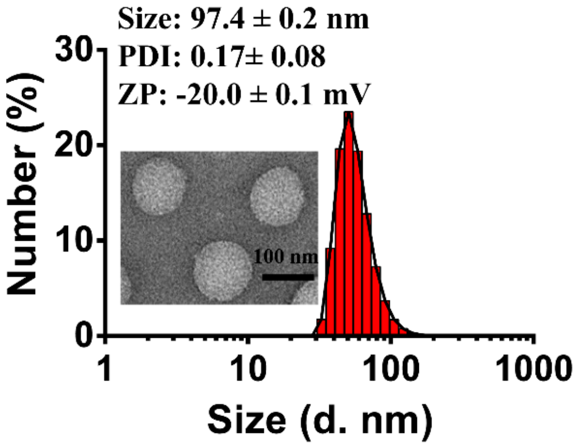

Figure S6. Morpology and size distribution of HCP determined by TEM and DLS. AD-SS-PPa (0.42 mg, 0.2 μmol) were dissolved in 100 μL of DMSO and added into 1 mL aqueous solution of HA-CD (0.4 mg, 0.20 μmol) during sonication under an ice bath. The purified HCP nanoparticles were obtained using dialysis with deionized water overnight (MWCO 3.5 kDa).

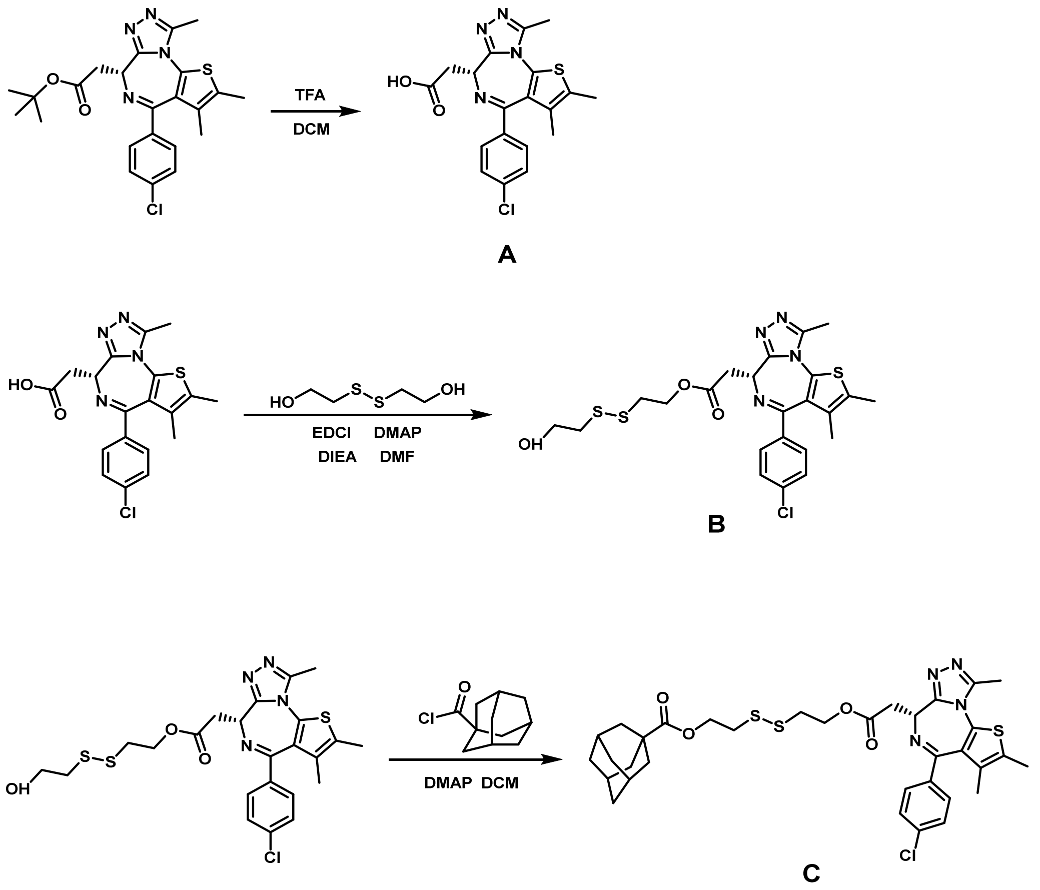

Figure S7. Synthetic route of JQ1-SS-AD.

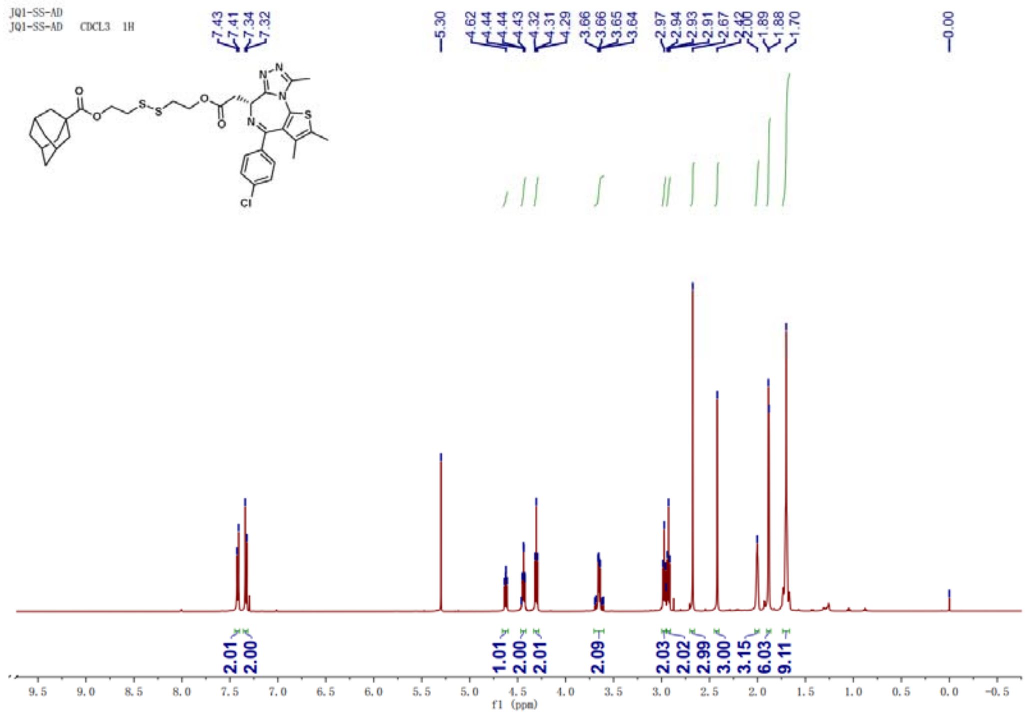

Figure S8. ^{1}H-NMR spectrum of AD-SS-JQ1.

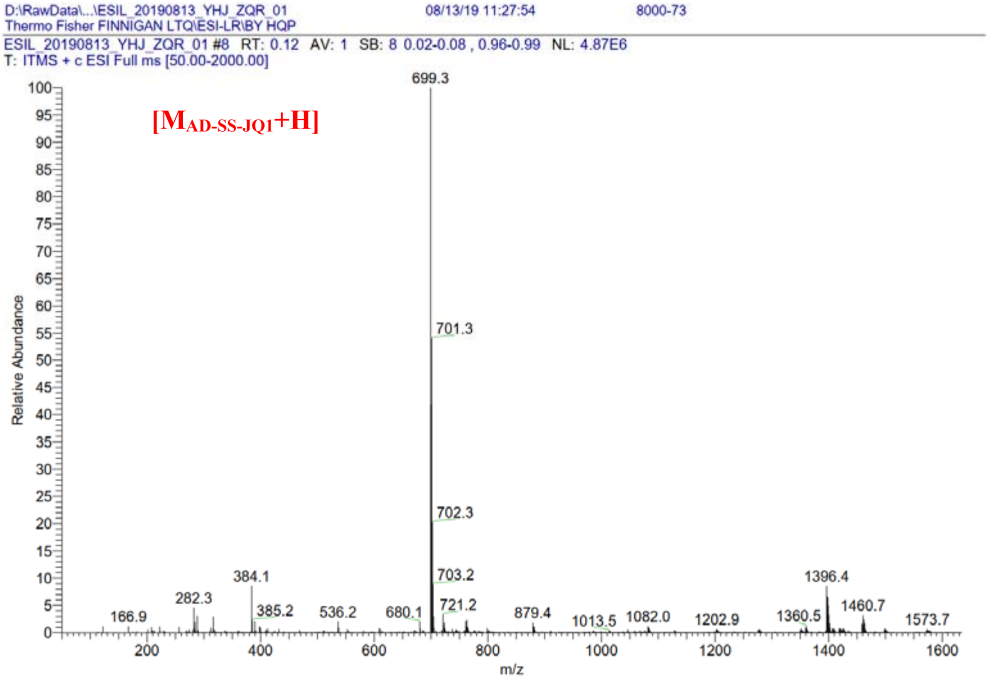

Figure S9. The ESI-MS spectrum of AD-SS-JQ1.

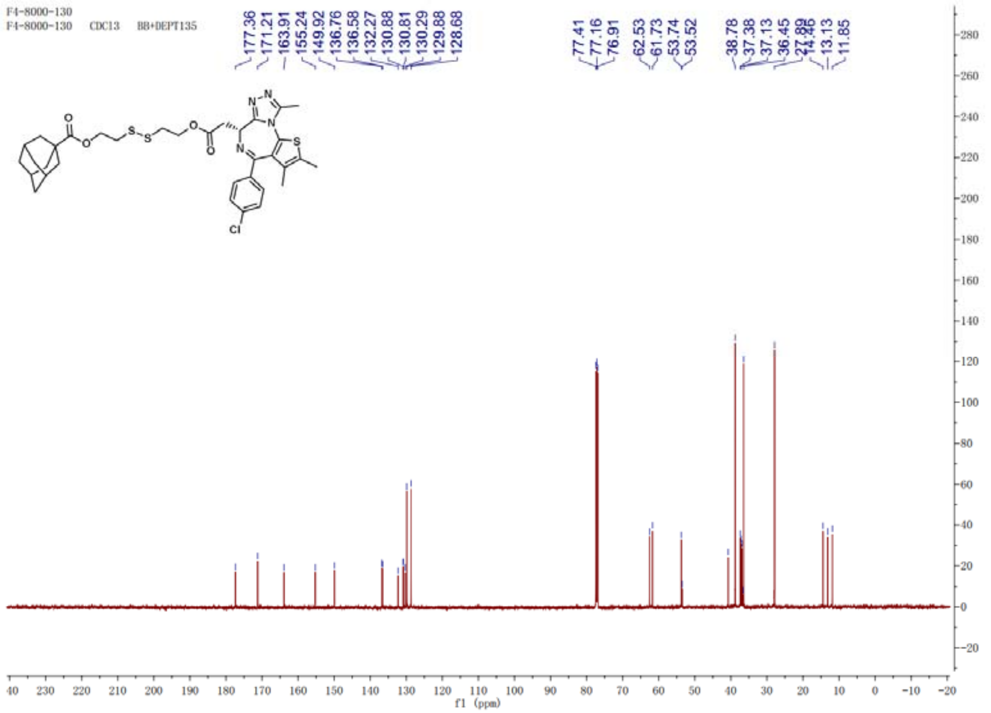

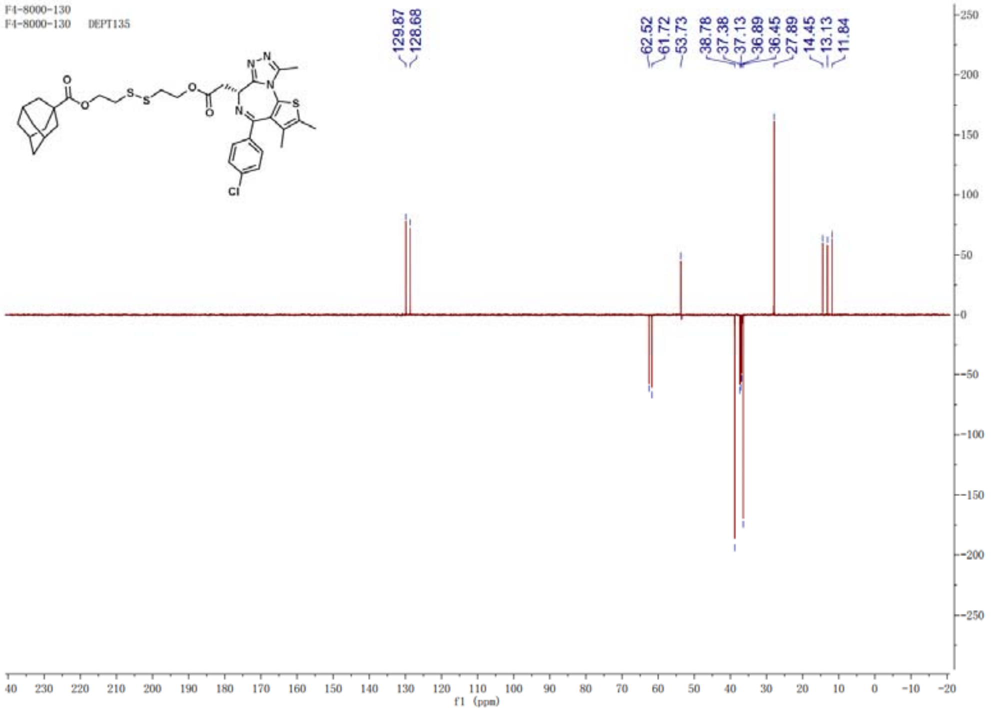

Figure S10. The ^{13}C NMR spectrum of AD-SS-JQ1.

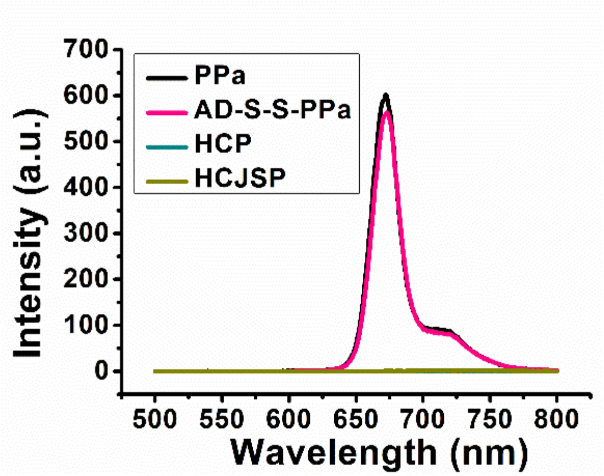

Figure S11. Fluorescence spectra of PPa, AD-SS-PPa, HCP, and HCJSP examined at an identical PPa concentration of 2.0×10^{-6} M (Ex = 415 nm).

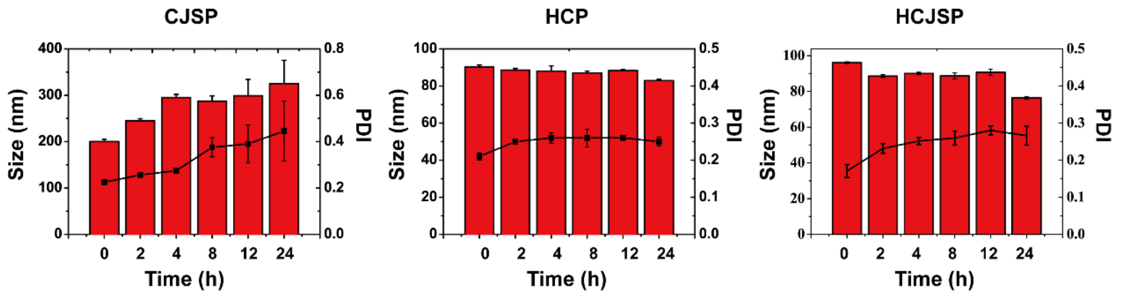

Figure S12. The colloidal stability of CJSP, HCP and HCJSP nanoparticles was analyzed in 10% fetal bovine serum by DLS.

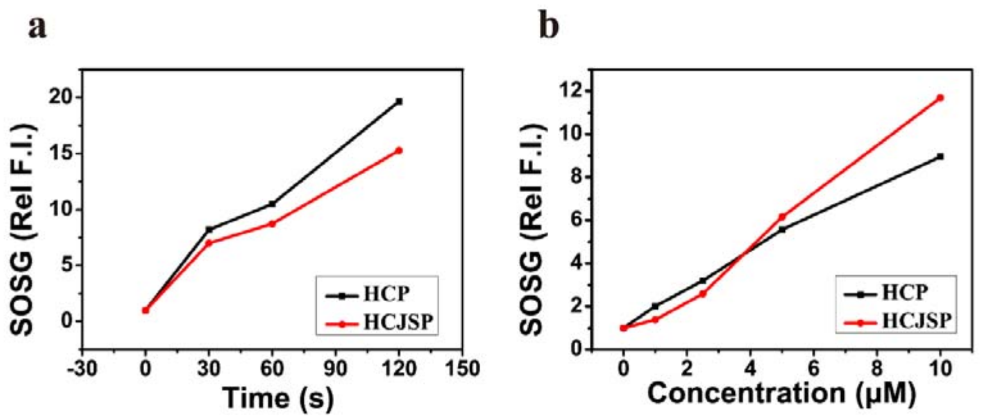

Figure S13. The prodrug nanoparticles in solution displayed photoactivity in irradiation (a) time- and (b) concentration-dependent manner. HCP and HCJSP solution upon 671 nm laser irradiation after being mixed with 10% SDS and 10 μM SOSG in 96-well black plates. PDT-induced ROS generation of the HCP and HCJSP was determined by microplate reader using SOSG as a ROS indicator (Ex/Em = 504/525 nm).

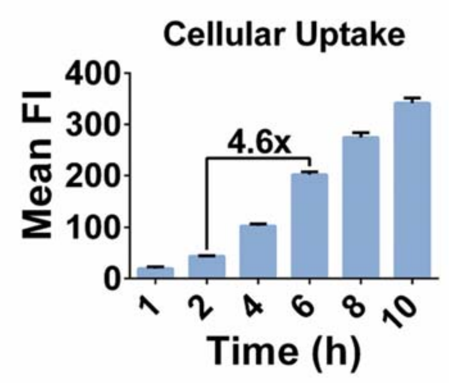

Figure S14. Flow cytometric analysis of intracellular uptake of the HCJSP nanoparticles in Panc02 tumor cells as a function of incubation time.

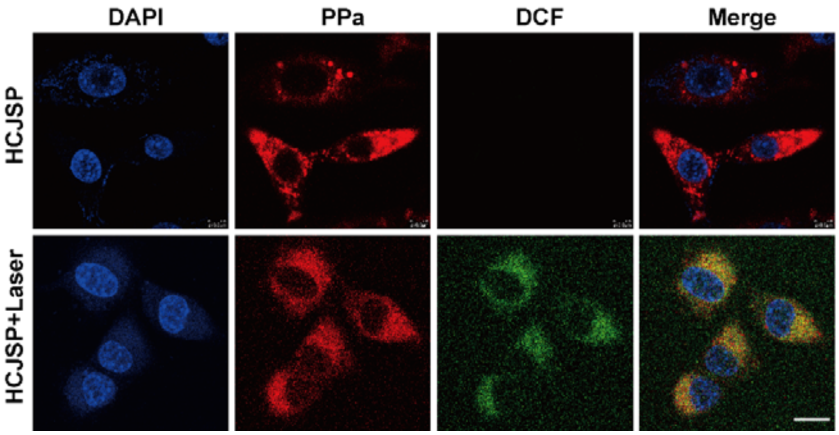

Figure S15. The representative CLSM images of intracellular ROS generation in Panc02 cells *in vitro*. Panc02 cells were incubated with HCJSP nanoparticles for 12 h. The cells were then irradiated with 671 nm laser for 30s at photodensity of 200 mW/cm^2 (scale bar = 25 μm).

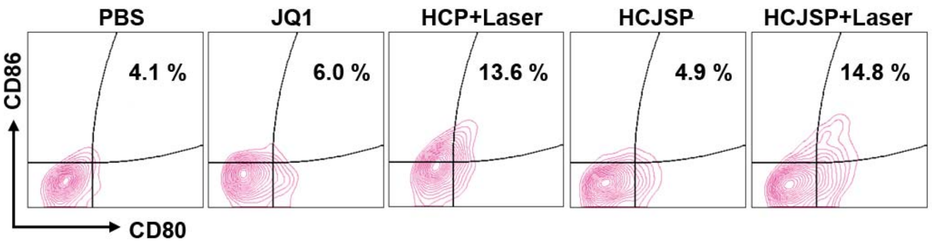

Figure S16. Representative flow cytometry plots of laser-induced BMDC maturation. The bone marrow-derived monocytes were extracted from C57BL/6 mice (6-8 weeks old) and maintained in complete RPMI containing mouse recombinant GM-CSF (20 ng/mL) and IL-4 (20 ng/mL) for 6-days to differentiate into immature BMDCs. The BMDCs were stimulated with tumor cells after indicated treatment for 24h, then collected and stained with fluorescence-labeled antibodies against CD11c, CD80, and CD86.

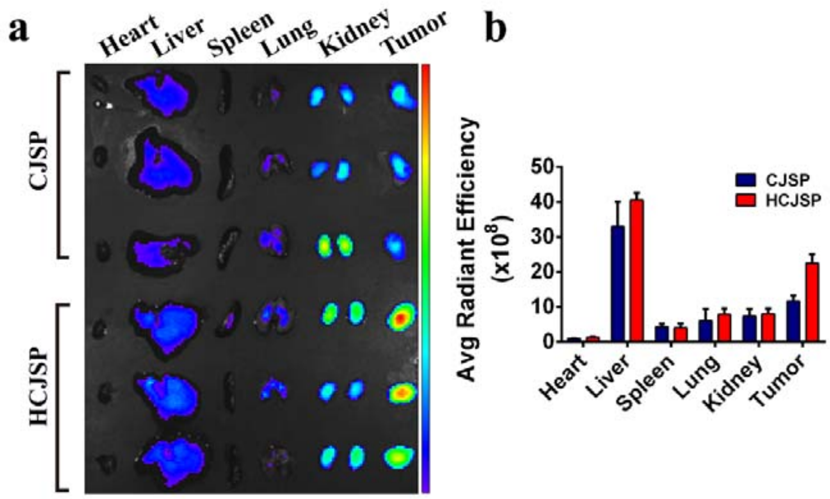

Figure S17. *Ex vivo* imaging and quantitative analysis of the CJSP and HCJSP distribution in the major organs and tumors of the Panc02 cells-bearing mice examined at 48 h post-injection (Ex/Em= 675/720 nm) (n = 3). Panc02 tumor-bearing mice were intravenously injected with PPa-loaded nanoparticles at a dose of 5 mg/kg.

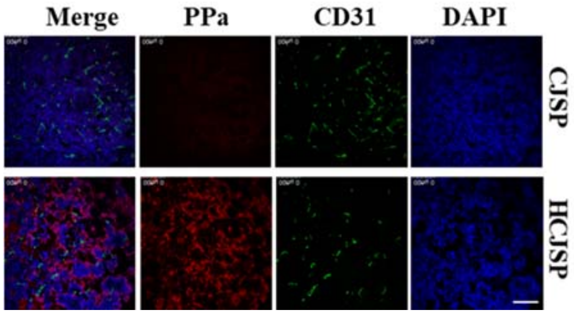

Figure S18. CLSM images of the HCJSP distribution in Panc02 tumor sections examined at 24 h post-injection. Panc02 tumor-bearing mice were intravenously injected with PPa-loaded nanoparticles at a dose of 5 mg/kg. Twenty-four h later, the tumors were collected to prepare the frozen sections and further stained with CD31-antibody (scale bar = 100 μm).

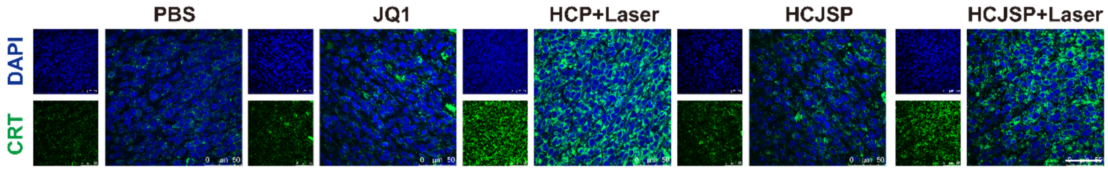

Figure S19. Immunofluorescence examination of CRT expression on the surface of the tumor cells *in vivo*. The mice were i.v. treated with JQ1, HCP or HCJSP at an identical PPa dose of 5.0 mg/kg or JQ1 dose of 15 mg/kg. The mice of the HCP+Laser and HCJSP+Laser group exposed to 671 nm laser irradiation (200 mW/cm^2) for 3 min at 24 h post-injection of prodrug nanoparticles. Twenty-four h later, tumors were harvested, fixed, and subjected to immunofluorescence staining with anti-CRT antibody (scale bar = 50 μm).

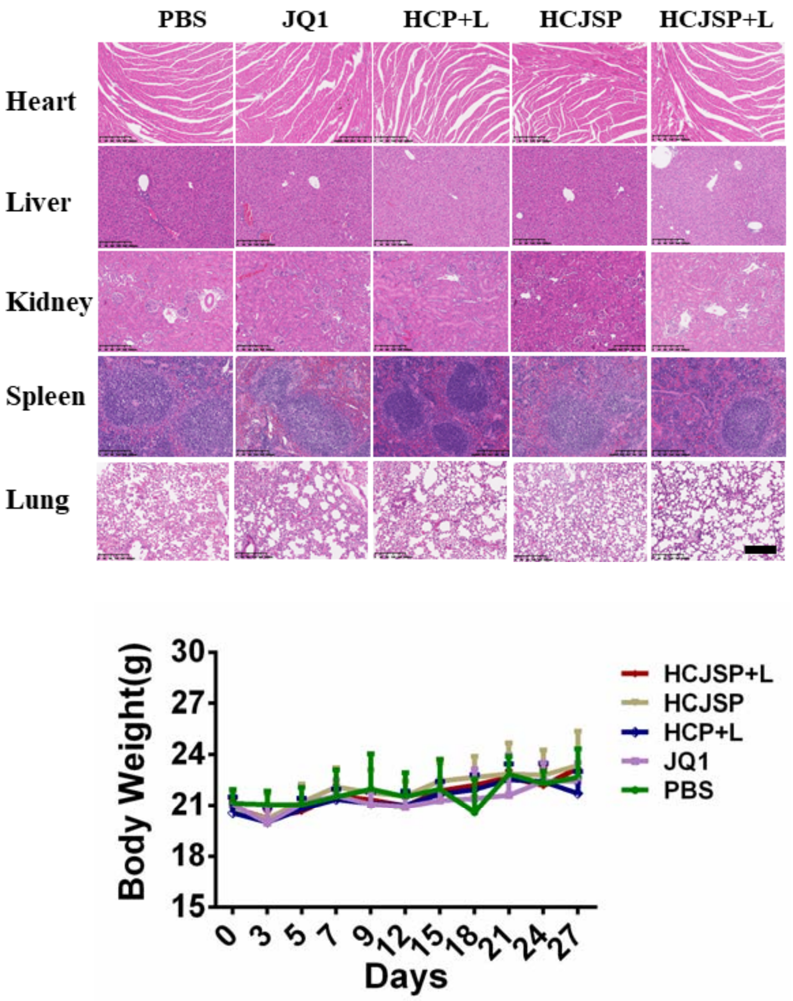

Figure S20. HE staining of the major organs and body weight change of Panc02 tumor-bearing mice following the indicated treatments (n = 5). All the major organs (heart, liver, kidney, spleen, and lung) were harvested at the end of the antitumor study. Histopathologic structure change and inflammatory cells infiltration of major organs were evaluated (scale bar = 200 μm).

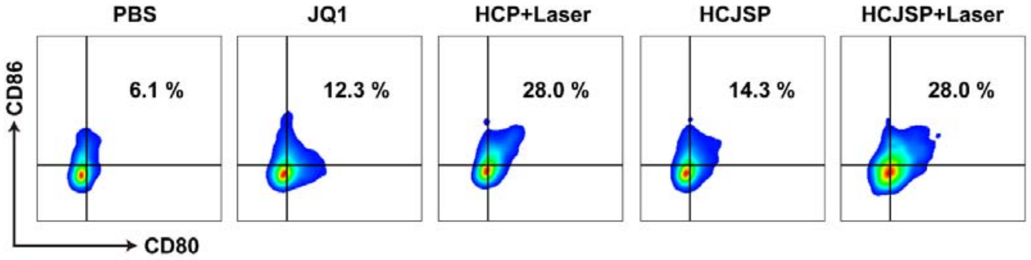

Figure S21. Flow cytometry of DC maturation (gated on $CD11c^+$) in the draining lymph nodes of Panc02 tumor-bearing C57BL/6 mice receiving different treatments (n = 3). The mice were i.v. treated with JQ1, HCP or HCJSP at an identical PPa dose of 5.0 mg/kg or JQ1 dose of 15 mg/kg. The tumors of HCP+Laser and HCJSP+Laser group received 671 nm laser irradiation (200 mW/cm^2) for 3 min at 24 h post-injection of prodrug nanoparticles. The draining lymph nodes were harvested and ground gently into a single-cell suspension. DC maturation marked by $CD11^+CD80^+CD86^+$ was determined by flow cytometry 7-days following indicated treatments.

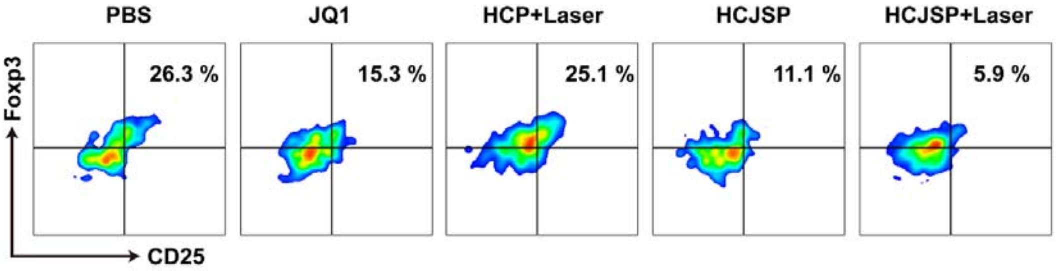

Figure S22. The intratumoral infiltration of Treg ($CD4^+CD25^+Foxp3^+$) in the Panc02 tumor-bearing C57BL/6 mice receiving different treatments (n = 3). Seven-days post the indicated treatments, the tumors were harvested and dissociated by the gentle MASCTM dissociator (Miltenyi, German). Tumor-infiltrating lymphocytes (TILs) were separated and enriched by mouse lymphocyte separation medium from the single-cell suspension. For detection of regulatory T cells subsets, the single cells suspension was stained with anti-CD3-PerCP-Cy5.5, anti-CD4-FITC, anti-CD25-APC and further with anti-Foxp3-PE pre-treated with permeabilization. All samples were incubated with specific antibodies for 30 min at 4 °C in the dark, then measured by FACS fortessa flow cytometry (Bioscience).

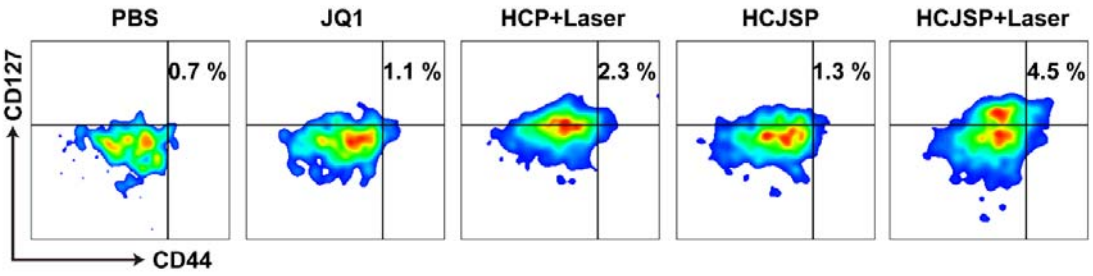

Figure S23. Flow cytometry analysis of memory T lymphocytes ($CD8^+CD44^+CD127^+$) in the spleen of Panc02 tumor-bearing C57BL/6 mice at 21-days post the final treatment (n = 3). The spleens were gently pressed with the piston of a syringe, and the single-cell suspension was obtained by 70 μm filtering membrane. Then the single cells were purified by erythrocyte lysis buffer and further stained with anti-CD8-FITC, anti-CD44-PerCP-Cy5.5 and anti-CD127-PE antibodies to determine the fraction of T_{EM} ($CD8^+CD44^+CD127^+$) by flow cytometry.

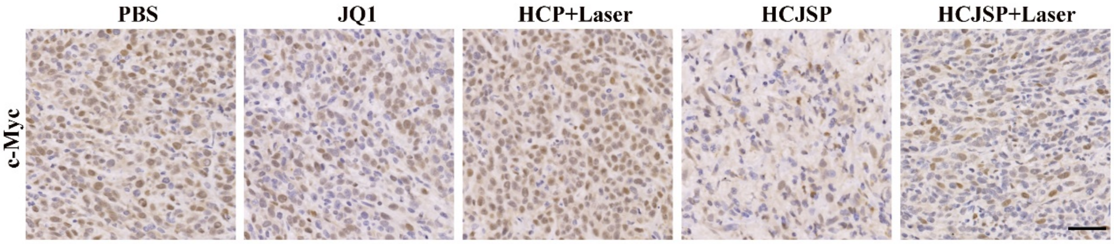

Figure S24. IHC examination of c-Myc expressions in the tumor tissue at the end of the antitumor study. Tumor tissues were harvested and fixed with 4% formalin at the end of the antitumor study. The tumor sections were subjected to immunohistochemical staining with an anti-*c*-*Myc* antibody (scale bar = 200 μm).
